# Optimising the prescribing of drugs that may cause dependency: An evidence and gap map of systematic reviews

**DOI:** 10.1177/13558196231164592

**Published:** 2023-05-29

**Authors:** Liz Shaw, Michael Nunns, Simon Briscoe, Ruth Garside, Malcolm Turner, GJ Melendez-Torres, Hassanat M Lawal, Jo Thompson Coon

**Affiliations:** 1 Research Fellow, Faculty of Health and Life Sciences, Exeter Policy Research Programme Evidence Review Facility, 3286 University of Exeter, UK; 2 Information Specialist, Faculty of Health and Life Sciences, Exeter Policy Research Programme Evidence Review Facility, 3286 University of Exeter, UK; 3 Associate Professor in Evidence Synthesis, Faculty of Health and Life Sciences, Exeter Policy Research Programme Evidence Review Facility and Faculty of Health and Life Sciences, European Centre for Environment and Health, 3286 University of Exeter, UK; 4 Patient and Public Representative, Faculty of Health and Life Sciences, Exeter Policy Research Programme Evidence Review Facility, 3286 University of Exeter, UK; 5 Professor in Clinical and Social Epidemiology, Faculty of Health and Life Sciences, Exeter Policy Research Programme Evidence Review Facility, 3286 University of Exeter, UK; 6 Graduate Research Associate, Faculty of Health and Life Sciences, Exeter Policy Research Programme Evidence Review Facility, 3286 University of Exeter, UK; 7 Professor of Evidence Synthesis and Health Policy – National Institute for Health and Care Research Applied Research Collaborations, Faculty of Health and Life Sciences, Exeter Policy Research Programme Evidence Review Facility, 3286 University of Exeter, UK

**Keywords:** drug-dependency, mapping review, adult

## Abstract

**Objectives:**

We set out to map the quantitative and qualitative systematic review evidence available to inform the optimal prescribing of drugs that can cause dependency (benzodiazepines, opioids, non-benzodiazepine hypnotics, gabapentinoids and antidepressants). We also consider how this evidence can be used to inform decision-making in the patient care pathway for each type of medication.

**Methods:**

Eight bibliographic databases were searched for the period 2010 to 2020. All included reviews were initially appraised using four items from the Collaboration for Environmental Evidence Synthesis Assessment Tool, with reviews that scored well on all items proceeding to full quality appraisal. Key characteristics of the reviews were tabulated, and each review was incorporated into an evidence and gap map based on a patient care pathway. The care pathway was based upon an amalgamation of existing NICE guidelines and feedback from clinical and patient stakeholders.

**Results:**

We identified 80 relevant reviews and displayed them in an evidence and gap map. The evidence included in these reviews was predominantly of low overall quality. Areas where systematic reviews have been conducted include barriers and facilitators to the deprescribing of drugs that may cause dependency, although we identified little evidence exploring the experiences or evaluations of specific interventions to promote deprescribing. All medications of interest, apart from gabapentinoids, were included in at least one review.

**Conclusions:**

The evidence and gap map provides an interactive resource to support (i) policy developers and service commissioners to use evidence in the development and delivery of services for people receiving a prescription of drugs that may cause dependency, where withdrawal of medication may be appropriate, (ii) the clinical decision-making of prescribers and (iii) the commissioning of further research. The map can also be used to inform the commissioning of further systematic reviews. To address the concerns regarding the quality of the existing evidence based raised in this report, future reviews should be conducted according to best-practice guidelines. Systematic reviews focusing on evaluating interventions to promote deprescribing would be particularly beneficial, as would reviews focusing on addressing the paucity of evidence regarding the deprescription of gabapentinoids.

## Introduction

Medications such as antidepressants, benzodiazepines, opioids, gabapentinoids and non-benzodiazepine hypnotics/z-drugs can or have been used to treat a variety of conditions such as anxiety, depression, insomnia and chronic pain.^1-4^ Approximately 26% of the adult population in England received a prescription for one or more of these medications in 2017–2018 and, whilst it is recommended these medications are prescribed for short-term use only, many patients take them beyond the short periods for which they are recommended.^5^ This appears to be an escalating problem, with prescription of these medications increasing over time. For example, within the UK, opioid prescription has more than doubled since 2018 and, for the people being prescribed opioids for musculoskeletal pain, it is estimated that nearly half are overprescribed.^6^

Prolonged use of some of these types of medications also places individuals at increased risk of physical dependence. For drugs such as opioids and benzodiazepines, patients risk becoming tolerant to the effects of the medication,^7,8^ or experience unpleasant withdrawal symptoms or side-effects,^9,10^ making it harder for them to reduce their medication use. Whilst use of antidepressants is not typically associated with the development of physical dependence as characterised by medication tolerance,^11^ the side-effects associated with withdrawal from antidepressants can be unpleasant, although these can usually be ameliorated (as with other types of drugs that may cause dependency) by a slow tapering process.^12^ However, some patients report feeling fearful they will be unable to remain well should they discontinue taking antidepressants and remain reluctant to trial reducing their dosage over time.^13^ Physical and psychological dependence have a potential cost-implication for health and social care services, both in terms of increasing numbers of prescriptions and in terms of resourcing services to support these patients. This represents a potential dilemma for prescribing clinicians, who must be aware of guidance to reduce the prescription of these medications, and also consider both the individual circumstances and preferences of individual patients during their decision-making.^14^

A greater understanding of the different factors that influence the prescription of drugs that may cause dependency from the perspectives of both prescriber and patient, may help to inform the design and delivery of services, clinical decision-making and commissioning of further primary or secondary research. There are a large number of existing systematic reviews of qualitative and quantitative evidence within this field. Hence, our aim was to create an evidence and gap map, a visual resource intended to display and present the quantity, quality and type of quantitative and qualitative systematic review evidence relevant to this topic and thus available to inform the optimal prescribing of drugs that may cause dependency.^15^

For the purposes of this review, we recognise that prescribing considers the wider system beyond the prescribing behaviour of an individual clinician, and requires knowledge of government laws, guidelines and policies and consideration of the individual views, experiences and needs of patients themselves. Thus, within this report the term ‘optimal prescribing’ encompasses both the prescribing behaviour of the clinician and the taking of medications by the patient.

Furthermore, we recognise that patient behaviour, with regard to taking their medications as prescribed, is ideally the product of an initial decision-making process between prescriber and patient, which is revised over time to consider factors such as effectiveness, side-effects and the extent to which medication taking can be incorporated into the patient’s daily routine. For convenience, we use the term ‘patient adherence’ throughout this report to encapsulate this continual decision-making process, in which the patient can choose, and/or be enabled, to play an active role. That said, interactions between patient and prescriber may not always be optimal, especially from the patient perspective.

We were interested in systematic reviews that synthesised the following:

- Evidence regarding the effectiveness or experiences of interventions intended to improve prescribing practices or patient adherence
- Evidence on the effectiveness or experiences of interventions intended to improve implementation of interventions intended to improve prescribing practices or patient adherence
- Evidence focusing on practitioner views or perceptions of making prescribing decisions
- Guidelines intended to inform prescribing practice.

The findings presented in this paper represent part of a broader mapping review, presented elsewhere, which provides further details about included studies and link to the evidence and gap map.^16^

## Methods

Development of the search strategy and the study selection and quality appraisal processes used in the creation of this evidence and gap map were consistent with those recommended by the University of York’s Centre for Reviews and Dissemination,^17^ with reference to Campbell Collaboration guidance for the development of evidence and gap maps.^15^ We registered a review protocol for this work.^18^ Our methods are summarised below according to PRISMA reporting guidance.^19^

### Search strategy

Eight bibliographic databases were searched on 11 August 2020: Cochrane Database of Systematic Reviews (via Cochrane Library), CINAHL (via EBSCO), EMBASE (via Ovid), Health Management Information Consortium (via Ovid), MEDLINE ALL (via Ovid), PsychInfo (via Ovid), Conference Proceedings Citation Index, and the Science and Science Citation Index (both via Web of Science, Clarivate Analytics). We also searched systematic review database Epistemonikos and preprint server medRxiv. These databases represent the outcome of considerable scoping and exploring on the most effective method to use to identify research relevant to the aims of this review.

Search terms for optimising prescribing were combined with terms for the medications of interest (benzodiazepines, z-drugs, opioids, gabapentinoids and antidepressants), with search strategies using both controlled headings and free-text searching, date limited from 2010 to 11 August 2020, with a systematic reviews study type filter. This date limit was selected to reflect the period relevant research published was likely to have been published within. Systematic reviews published outside this period would be considered out of date. In addition, we undertook backwards citation chasing for all reviews that met our inclusion criteria, searched topically relevant websites and pursued full texts for conference abstracts and review protocols identified through our searches, contacting authors of relevant articles where full texts could not be found. An example search strategy and list of websites searched can be found in the online supplement, with our full search strategy reported in the main project report.^16^ At the time of publication, searches were over 1 year old, thus we completed updated searches in MEDLINE ALL (via OVID) on 2 August 2022. We restricted our update searches to MEDLINE as this reflected that all the studies included from our original search were indexed in this database.

This approach draws upon existing theory to streamline the search update process and reflects that all the studies included from our original search were indexed in MEDLINE.^20^ This focused search allowed us to expedite the update process whilst ensuring we searched for recent literature where it would most likely be indexed.

### Study selection

Following an initial calibration exercise where a sample of 100 titles and abstracts identified through bibliographic database searches were double screened by two reviewers (LS, MN or HL), the revised inclusion criteria (see Table 1) were then applied independently by two reviewers to each identified citation (LS, MN or HL), with disagreements resolved through discussion or referral to a third reviewer (SB). Eligibility of full texts was assessed using the same method. Screening decisions were recorded in Endnote X8 software (Clarivate Analytics, Philadephia, PA, USA) and the study selection process was detailed within a PRISMA-style flowchart.

**Table 1.** Inclusion criteria for review.

General criteria	Inclusion criteria
Population	Mean age ≥16 Prescription for one or more of the following medications is being considered or already being received: - Benzodiazepines - Non-benzodiazepine hypnotics (e.g. z-drugs) - Opioids - Antidepressants - Gabapentinoids
Intervention/Phenomenon of interest	For systematic reviews of quantitative evidence, interventions at system, prescriber or patient levels which aimed to improve one or more of the following: - Adherence to prescribed medication of interest - Prescriber adherence to clinical guidance for prescribing - Prescriber practices - Implementation of an intervention to enhance patient adherence or prescriber practices. For systematic reviews of qualitative evidence, review foci could include the views or experiences of patients, carers or clinicians for: - Healthcare consultations to discuss initiation, reviewing or discontinuing a prescription - Interventions to improve adherence/prescribing practice - Reasons for adherence or non-adherence to prescribed medication - Making prescribing decisions
Comparator/Context	Any
Outcome	Reviews were relevant if their outcomes were consistent with the above aims and stated in the abstract
Study design	Systematic reviews which met the following criteria as outlined by Martinic et al. (2019):^16^ - Clearly stated research question - Indicate which sources were searched - Reproducible search strategy and search date - Clearly defined inclusion and exclusion criteria and study selection methods - Critically appraise and report the quality of included studies - Reproducible data synthesis strategy. Encompasses: systematic reviews of quantitative and qualitative literature, systematic reviews of reviews, systematic reviews of guidelines, scoping and rapid reviews
Language restriction	None

### Patient care pathways

Patient care pathways, for antidepressants and for other drugs that may cause dependency (benzodiazepines, opioids, hypnotics/z-drugs and gabapentinoids), were developed to act as the basis for the evidence and gap map. The initial pathways were based upon the NICE guidelines for each of the medications of interest and for optimising prescribing.^2,21-22^ We supplemented the guidance with information from other relevant publications.^5,23^ To ensure the pathways reflected not only best practice, but also how patients and their family and carers could interact with the pathway, we also drew upon a systematic review of qualitative evidence that explored patient experiences of medication taking.^24^ Following consultation with our stakeholders, a condensed version of the map was then produced, which focused on four key decision points on this pathway: pre-treatment/initiation, maintaining treatment, discontinuation of treatment and guidelines. More details on our stakeholder consultation can be found in the online supplement.

### Data extraction and quality appraisal

Following an initial pilot (*n* = 5 papers) by two reviewers (LS, MN), the data extraction form was applied to all included systematic reviews by one reviewer (MN or LS) and checked by a second (SB, LS or MN) with any disagreements settled through discussion or referral to a third person. Data extracted included key bibliographic information, characteristics of the review and included participants, and information on which part of the patient care pathway could be informed by the review.

The extent to which the studies met the criteria for a systematic review as defined within our inclusion criteria varied considerably, particularly with regard to the rigour and/or transparency of reporting of methods of searching and critical appraisal. To highlight the most robust evidence within the evidence and gap map, a deviation from the protocol was made to extend the eligibility criteria to focus more resource on the reviews with more robust methods. Firstly, two reviewers (LS, MN) independently applied four modified criteria from the Collaboration for Environmental Evidence Synthesis Assessment Tool (CEESAT) to each study eligible for inclusion in the review.^25^ Disagreements were resolved through discussion. The criteria were:

1. Search strategy: Is the approach to searching clearly defined, systematic and transparent?
2. Is the search comprehensive?
3. Does the review critically appraise each study?
4. During critical appraisal was an effort made to minimise subjectivity?

Only systematic reviews that met all four criteria for robustly conducted systematic reviews were then prioritised for full quality appraisal using a version of the AMSTAR-2, modified to include reporting standards for qualitative evidence synthesis.^26^ Reviews that did not meet all four CEESAT criteria were awarded the equivalent of a ‘Critically Low’ quality rating on the AMSTAR-2.

Data extraction and quality appraisal were conducted using EPPI-Reviewer (v 4.11.5.2). A full list of data extracted from each review, including the amended CEESAT and AMSTAR-2 quality appraisal criteria, can be found in the online supplement.

### Data analysis and presentation

The following details were tabulated for all included reviews: review author, date of publication, indicator of study quality if applicable, focus of review, type of primary studies included, review synthesis methods, eligible age of participants, medication of interest and relevance to the aim of the systematic mapping review. For quantitative studies, a table outlining the aims, features and outcomes of the intervention being evaluated, and relevant parts of the patient care pathway was constructed. The same details were tabulated for qualitative systematic review evidence and Clinical Practice Guidelines (CPGs), although intervention details were replaced with information regarding the perspectives of participants obtained and phenomenon of interest or a description of the CPG aim. Details of the systematic reviews identified through update searches are provided in the online supplementary materials.

### Evidence and gap map

We used EPPI-Mapper software (v 1.2.5) to organise the included systematic reviews according to the medication of interest and relevant part of the patient care pathway.^27^ This produced a grid, which formed the basis of our evidence and gap map, which is available via the online supplement. Within each segment of the map, systematic review evidence is presented according to the ‘Overall Quality’ rating provided by the AMSTAR-2. The colour and size of the bubbles indicate the quality rating and quantity of available evidence for that medication type at that part of the care pathway respectively. Systematic reviews relevant to more than one type of medication, or relevant to more than one position on the care pathway are represented within more than one segment of the map. The type of evidence shown in the map can be altered by using a variety of filters, based on the data extracted from each review (see online supplement). Viewers can click on the map to view the abstracts of each systematic review included within each segment.

## Results

Following de-duplication, 2729 records were screened at title and abstract level. Of these, 2492 records were excluded, leaving 235 records to screen at full text, of which 160 were later excluded. To the total of 78 included systematic reviews, an additional two eligible reviews were found through the search strategy associated with the second strand of the wider mapping review (which included statins) on which this paper is based.^11^ A link to the evidence and gap map, displaying the main features of included reviews identified through the original searches, can be accessed via on the online supplement. Details of the two reviews identified through update searches are provided within the online supplement. This is because they were both of Critically Low quality and represented segments of the map where there are already many existing reviews. In addition, each of these reviews only contained 1 study relevant to the aims of our review.

The PRISMA diagram summarising how all included reviews were identified is provided in the online supplement. A summary of the characteristics of the reviews included in the map is provided below, with full details in the online supplement. Below, the reviews are cited as S1 to S80, corresponding to their enumeration in the online supplement.

### Review characteristics

Of the 80 reviews eligible for inclusion, 77 described themselves as systematic reviews, with another two describing themselves as ‘scoping reviews’ (see supplemental material s46, s62) and the final one as a ‘rapid review’ (see Supplemental material s39). Medications of interest within each review included antidepressants (*n* = 24) (see Supplemental material s2, s7, s9, s11–s13, s21, s24, s25, s26, s33, s34, s38, s44, s45, s48, s49, s54, s56, s60, s64, s65, s68, s73), a mixture of drugs that may cause dependency (*n* = 24) (see supplemental material s3,s5,s8,s10,s14,s18,s28,s29,s35,s39,s41,s42,s50,s51,s53,s57,s58,s59,s61-s63,s66,s67,s70), opioids (*n* = 24) (see supplemental material s4,s6,s15,s19,s20,s22,s23,s30,s31,s36,s37,s43,s46,s47,s55,s71,s72,s74–s80), benzodiazepine (*n* = 7) (see supplemental material s16,s17,s27,s32,s40,s52,s69), hypnotics/z-drugs (*n* = 1) (see supplemental material s1) and gabapentinoids were not represented in any of the included reviews.

Eligible age was reported within the inclusion criteria of each review as follows: people 16 or 17 or over (*n* = 3) (see supplemental material s9,s17,s65), 18 or over (*n* = 21) (see supplemental material s2,s5,s11,s18,s19,s22,s24,s25,s34,s37,s42-s46,s49,s56,s69,s71,s77,s80), people 60 or 65 years or older (*n* = 7), (see supplemental material s1,s14,s27,s29,s58,s66,s52), adults unspecified (*n* = 6) (see supplemental material s28,s30,s33,s47,s55,s72) or patients of any age (*n* = 1) (see supplemental material s13). Thirty-four reviews did not explicitly state the age of the populations of eligible studies within their inclusion criteria (NR) (see supplemental material s3,s4,s6,s7,s10,s12,s15,s16,s20,s21,s23,s26,s31,s32,s36,s39,s38,s46,s48,s51,s53,s54,s57,s59,s60,s61,s63,s68,s70,s73,s74,s75,s76,s78), and in 9 reviews, the population age was categorised by the review team as ‘Other’ (see supplemental material s8,s32,s33,s35,s40,s41,s50,s64,s67).

Of the 80 reviews, 23 were fully quality appraised using the modified AMSTAR-2. Eight were of ‘High’ overall quality (see supplemental material s1,s5,s9,s19,s26,s59,s63,s79), 10 were of ‘Moderate’ overall quality (see supplemental material s27,s32,s40,s41,s43,s44,s49,s54,s71,s80) and five were of ‘Low’ overall quality (see supplemental material s18,s24,s35,s4,s45). The remaining 56 reviews did not meet the four criteria from the CEESAT and were thus categorised as ‘Critically Low’ quality on the AMSTAR-2 scale. Thirty of these reviews scored poorly on one CEESAT item (see supplemental material s2,s4,s6,s7,s12,s15,s17,s20,s22,s25,s30,s31-s33,s38,s48,s50,s51,s56,s57,s58,s65,s66,s68,s37,s55,s69,s75–s77) with the remaining 26 scoring poorly on between 2 and 4 of the CEESAT criteria (see supplemental material s3,s8,s10,s11,s14,s16,s21,s23,s28,s29,s34,s36,s39,s46,s47,s52s,s53,s60,s61,s62,s64,s67,s70,s72,s73,s74). Scores of the included reviews on the modified AMSTAR-2 tool and CEESAT criteria can be viewed in the online supplement.

A summary of the foci of the 78 systematic reviews included in this evidence and gap map is provided below in Table 2.

**Table 2.** Summary of foci of systematic review evidence included in evidence and gap map.

Topic [Type of evidence]	Number of reviews^a^	Appraised using AMSTAR-2 Y/N: n (Overall Quality rating)	Medication of interest
Prescriber, patient and/or family/carer views of issues relating to prescribing and/or adherence to medications of interest [Qualitative]	11^b^	Y: 1 (Moderate)	AD: 1
		N: 10	AD: 2 AD+DCD: 3 BZ: 1 BZ+AD: 1 DCD Mix: 1 Opioids: 2
Evaluating intervention to optimise prescribing [Quantitative]	26	Y: 8 (High: 2, Moderate: 5, Low: 1)	AD: 2 AD+DCD: 1 BZ+Hypnotics/z-drugs: 2 Opioids: 3
		N: 19	AD: 3 DCD+AD: 3 BZ: 1 DCD Mix: 1, Opioids: 11
Evaluating intervention to deprescribe medication [Quantitative]	22	Y: 9 (High: 3, Moderate: 3, Low: 2, Critically Low: 1)	AD: 1 BZ: 2 DCD+AD: 1 DCD Mix: 2 Hypnotics/z-drugs: 1 Opioids: 2
		N: 13	BZ: 3 DCD+AD: 2 DCD Mix: 5 Opioids:3
Evaluating intervention to optimise patient adherence to a medication [Quantitative]	18	Y: 4 (High: 2, Moderate:1, Low: 1)	AD: 3 DCD+AD: 1,
		N: 15	AD: 13 BZ: 1 DCD+AD: 1
Recommendations/clinical practice guidelines regarding prescribing of opioids for pain (4 chronic, 2 acute following surgery) [Guidelines]	6	Y: 1 (High)	Opioids: 1
		N: 5	Opioids: 5

AH = Antihypertensive; AD = Antidepressant; BZ = Benzodiazepines; DCD = Drugs which may Cause Dependency; N = No; n = Number; Y = Yes.

^a^Number > 78 as some reviews had multiple aims.

^b^Two reviews within qualitative category also synthesised quantitative data.^S60,S73^

### Summary of evidence on the patient care pathway

The number of systematic reviews relevant to each part of the patient care pathway, according to medication of interest is summarised in Table 3.

**Table 3.** Number of reviews and their quality at each stage of patient care pathway.

Medication of interest	Care pathway (N: High, Moderate, Low, Critically Low quality)			
	Pre-treatment/initiation	Maintaining treatment	Discontinuing treatment	Prescribing guidelines
Benzodiazepines	14 (1,1,0,12)	6 (0,0,0,6)	28 (1,3,3,21)	1 (0,0,0,1)
Opioids	23 (2,2,1,18)	5 (1,0,0,4)	15 (1,2,1,11)	7 (1,0,0,6)
Hypnotics/z-drugs	7 (1,1,0,5)	3 (0,0,0,3)	16 (2,1,3,10)	0
Antidepressants	21 (3,2,0,16)	23 (3,1,1,18)	14 (0,2,1,11)	1 (0,0,0,1)

Below we present a summary of the evidence provided within each segment of the evidence and gap map based on the foci and/or aims of interest of the original review, organised by medication of interest.

### Benzodiazepines

#### Pre-treatment/initiation

Of the 14 reviews relevant to the ‘pre-treatment/initiation’ stage of the patient care pathway with benzodiazepines as the medication of interest, four were qualitative studies of ‘Critically Low’ quality, two including the prescriber perspective (see supplemental material s14,s69), one perspective of patients (see supplemental material s70) and one where the perspective was unclear (see supplemental material s28). Eight reviews coded at this part of the pathway evaluated interventions to optimise the prescribing of medication, one of ‘High’ quality (see supplemental material s59), one of ‘Moderate’ quality (see supplemental material s41) and six of ‘Critically Low’ quality (see supplemental material s8,s10,s28,s39,s52,s53).

Two reviews of ‘Critically Low’ quality that evaluated interventions within this section of the pathway had multiple aims (see supplemental material s61,s16). These aims included improving adherence to medication, and benzodiazepines were one type of medication included within these studies. One review focused on the impact of Medicare Part D on under/overuse of various medications, and so was only relevant within the US setting (see supplemental material s61) and the other focused on collating primary studies to develop a community based pharmacy intervention for people affected by dementia (see supplemental material s16). These studies were also placed within maintaining and discontinuation sections of the care pathway. Finally, two ‘Critically Low’ quality reviews focused on interventions whose aims encompassed promoting the reduction in use of medications, which included benzodiazepines (see supplemental material s16, s29).

#### Maintaining treatment

Three of the six reviews included in this segment synthesised qualitative evidence, one focusing on the perspectives of patients (see supplemental material s70), one on the views of prescribers (see supplemental material s3) and one where perspective was unreported (see supplemental material s28). Two reviews synthesised primary evidence that evaluated interventions to optimise prescribing (see supplemental material s10, s28), one review focused on the impact of Medicare part D (an insurance programme for prescription medication) on over- and under-use of medications (see supplemental material s61) and one review focused on synthesising evidence to inform the development of a community pharmacist intervention for people with dementia (see supplemental material s16).

#### Discontinuing treatment

Five of the 28 reviews within this segment synthesised qualitative evidence, all were of ‘Critically Low’ quality (see supplemental material s3,s14,s67,s69,s70). Two included patient and/or carer perspectives (see supplemental material s67, s70) and three focused on the experiences of prescribers (see supplemental material s3, s14, s69). Eight reviews evaluated quantitative evidence that focused on optimising the prescribing of medications overall quality included ‘Moderate’ (*n* = 1) (see supplemental material s41 ‘Low’ (*n* = 1) (see supplemental material s35) and ‘Critically Low’ (*n* = 6) (see supplemental material s8, s10, s28,s39, s42, s50).

Fifteen reviews evaluated interventions that focused on deprescribing. One was ‘High quality’ (see supplemental material s5), two ‘Moderate’ quality (see supplemental material s40,s52), two ‘Low’ quality (see supplemental material s18,s42) and 10 ‘Critically Low’ quality (see supplemental material s16,s,17,s29,s50,s51,s57,s58,s62,s66,s69.

#### Prescribing guidelines

One ‘Critically Low’ quality review of quantitative evidence explored the impact of CPGs on prescribing practice in mental health settings (supplemental material s53).

### Opioids

#### Pre-treatment/initiation

Three ‘Critically Low’ quality systematic reviews of the 23 reviews relevant to the prescription of opioids conducted a qualitative synthesis of prescriber views (see supplemental material s14,s36,s72). 17 reviews synthesised quantitative evidence evaluating interventions to optimise the prescribing of medications that included opioids. Quality of these reviews were as follows ‘Moderate’ (*n* = 2) (see supplemental material s71,s80), ‘Low’ (*n* = 1) (see supplemental material s76) and ‘Critically Low’ (*n* = 14) (see supplemental material s4,s6,s15,s20,s23,s30,s31,s37,s39,s46,s47,s74,s75,s77). One ‘High’ quality review synthesised evidence relating to the effectiveness of automated telephone communication systems for preventative health care and management of long-term conditions (see supplemental material s29). Three ‘Critically Low’ quality reviews synthesised quantitative evidence relating to the deprescribing of opiods (see supplemental material s46,s29,s23).

#### Maintaining treatment

Three ‘Critically Low’ quality reviews synthesised qualitative evidence exploring prescriber views on prescribing opioids (see supplemental material s3,s36,s72). One ‘High’ quality review synthesised quantitative evidence evaluating telephone communications, one outcome of which was impact on adherence to medication (see supplemental material s63). One ‘Critically Low’ quality study reviewed the association of state opioid misuse prevention policies on patient and provider outcomes (see supplemental material s46).

#### Discontinuing treatment

Three ‘Critically Low’ quality reviews synthesised qualitative evidence of prescriber experiences (see supplemental material s3,s14,s72). Eight reviews synthesised quantitative evidence valuating interventions to optimise the prescribing of medication, two were ‘Moderate’ quality (see supplemental material s43,s71), one of ‘Low’ quality (see supplemental material s76) and five of ‘Critically Low’ quality (see supplemental material s4,s23,s39,s46,s55). Seven reviews evaluated the effectiveness of interventions aiming to deprescribe medications, including opioids. Overall quality ratings were: ‘High’ (*n* = 1) (see supplemental material s19), ‘Moderate’ (*n* = 1) (see supplemental material s43) and ‘Critically Low’ (*n* = 5) (see supplemental material s22,s23,s29,s46,s57).

#### Prescribing guidelines

Six reviews of ‘Critically Low’ quality synthesised Clinical Practice Guidelines focusing on optimising prescribing of opioids (see supplemental material s6,s15,s20,s30,s47,s55).

### Hypnotics/z-drugs

#### Pre-treatment/initiation

One ‘Critically Low’ quality review synthesised qualitative evidence regarding patient views of seeking treatment (see supplemental material s70) and five reviews synthesised quantitative evidence on the effectiveness of interventions to optimise prescribing. Overall quality was as follows: ‘High’ (*n* = 1) (see supplemental material s59), ‘Moderate’ (*n* = 1) (see supplemental material s41) and ‘Critically Low’ (*n* = 3) (see supplemental material s8,s28,s52). One ‘Critically Low’ quality review focused on medication that reduced the risk of the patient falling (see supplemental material s29).

#### Maintaining treatment

Three ‘Critically Low’ quality reviews synthesised qualitative evidence regarding maintenance of hypnotic treatment. One reported patient views, supplemental material s70 one reported prescriber views supplemental material s3 and one did not clearly report perspectives sought (see supplemental material s28). This latter ‘Critically Low’ quality review evaluated evidence regarding the optimal prescribing of hypnotics/z-drugs (see supplemental material s28).

#### Discontinuing treatment

Three ‘Critically Low’ quality reviews synthesised qualitative evidence (see supplemental material s3,s28,s70), including patient (see supplemental material s70), prescriber (see supplemental material s3) and not-reported views (see supplemental material s28). Six reviews synthesised quantitative evidence evaluating interventions to optimise the prescribing of medications of interest. One was of ‘Moderate’ quality (see supplemental material s41), one of ‘Low’ quality (see supplemental material s35) and four of ‘Critically Low’ quality (see supplemental material s8,s28,s51,s52). Nine reviews evaluated interventions aiming to deprescribe the medications of interest (Overall quality: ‘High’ (*n* = 2) (see supplemental material s1,s5), ‘Low’ (*n* = 2) (see supplemental material s18,s42) and ‘Critically Low’ (*n* = 5) (see supplemental material s29,s62,s50,s51,s66).

#### Prescribing guidelines

No reviews of hypnotics/z-drugs considered this stage of the patient care pathway.

### Antidepressants

#### Pre-treatment/initiation

Four ‘Critically Low’ quality reviews synthesised qualitative evidence, where the perspectives obtained included prescriber (see supplemental material s14,s21), patient/prescriber/carer views (see supplemental material s64) and not reported (see supplemental material s28). 11 reviews included quantitative evidence evaluating evidences aiming to optimise prescribing of medication. One was rated as ‘High’ quality (see supplemental material s26), two as ‘Moderate’ quality (see supplemental material s41,s49) and eight as ‘Critically Low quality (see supplemental material s7,s8,s10,s28,s38,s39,s53,s73). Seven reviews included evidence evaluating interventions where the primary or secondary aim was to improve adherence. One was rated as ‘High quality’ (see supplemental material s63) the remainder ‘Low’ quality (see supplemental material s9,s38,s48,s56,s60,s61). One ‘Critically Low’ quality review focused on use of drugs that induced the risk of falls in older adults (see supplemental material s29).

#### Maintaining treatment

Three ‘Critically Low’ quality reviews synthesised qualitative evidence regarding patient views (see supplemental material s3), patient and prescriber views (see supplemental material s64) or not reported (see supplemental material s28). Five reviews synthesised quantitative evidence evaluating interventions to optimise the prescribing of medications, including antidepressants. Overall quality included one ‘High’ quality review (see supplemental material s26) and four ‘Critically Low’ quality reviews (see supplemental material s38,s73,s10,s28).

Seventeen reviews synthesised quantitative evidence evaluating interventions to promote medication adherence. Two were of ‘High’ quality (see supplemental material s9,s63), one of ‘Moderate’ quality (see supplemental material (see supplemental material s54), one of ‘Low’ quality (see supplemental material s24) and 13 of ‘Critically Low’ quality (see supplemental material s2,s11,s12,s25,s33,s34,s38,s48,s56,s60,s61,s65,s68).

#### Discontinuing treatment

Five were reviews of qualitative evidence. One review of patient experiences was rated as ‘Moderate’ quality (see supplemental material s44). The remaining four were of ‘Critically Low’ quality of qualitative evidence (see supplemental material s3,s14,s28,s67), two seeking prescriber views (see supplemental material s3, s14) and one patient views (see supplemental material s67). One review did not report perspective sought (see supplemental material s28).

Five reviews focused on evaluating interventions aiming to optimise the prescribing of medication. Overall quality included ‘Moderate’ (*n* = 1) (see supplemental material s41) and ‘Critically Low’ (*n* = 4) (see supplemental material s39,s8,s10,s28). One ‘Critically Low’ quality review synthesised evidencing evaluating interventions to promote adherence to range of medications (see supplemental material s61). Four reviews synthesised evidence promoting the deprescribing of medications, one of ‘Low’ quality (see supplemental material s18) and three of ‘Critically Low quality (see supplemental material s45,s29,s58).

#### Prescribing guidelines

One ‘Critically Low’ quality review synthesised evidence evaluating impact of CPGs on prescribing in mental health (see supplemental material s53).

#### Summary of evidence and gaps

The only medication of interest not represented by any systematic review evidence within the map was gabapentinoids. The part of the care pathway with the most evidence mapped to it was the ‘discontinuing treatment’ stage, where the majority of evidence was regarding benzodiazepines. There was also a cluster of evidence evaluating interventions to optimise the prescribing of opioids, although all medications (apart from gabapentinoids) were represented within at least one systematic review with this focus. However, the quality of this systematic review evidence was variable, with the majority of the evidence being of ‘Critically Low’ quality.

In comparison to the quantity of quantitative systematic review evidence, the volume of qualitative reviews included in the map was more limited. The included qualitative reviews tended to focus on the general barriers and facilitators to the deprescribing of drugs that may cause dependency and antidepressants, rather than exploring experiences or views of specific interventions to optimise prescribing. Overall, the volume of evidence relevant to the prescribing of non-benzodiazepine hypnotics or z-drugs was much lower than for the other types of medication. Systematic reviews of guidelines focused on the use of opioids to manage pain.

## Discussion

The evidence map indicates areas where greater consideration is required when considering if funding further reviews will be beneficial, particularly when synthesising evidence that evaluates interventions intending to promote the optimisation or deprescribing of drugs that may cause dependency. Whilst high numbers of systematic reviews exist in these areas, many are of low quality, which may limit the extent they are useful to inform policy. Reviews of critically low quality failed to meet all four of the CEESAT criteria used within the initial stage of quality appraisal.^25^ That is, they did not conduct or report details one of more of the following: a replicable *and* comprehensive search, quality appraisal highlighting scores for every included study on each individual quality appraisal item, and/or a process to minimise reviewer bias during the quality appraisal process. The low quality of the majority of the included systematic reviews limits the confidence that can be placed in their findings and their use to commissioners and policy makers in their decision-making.

The evidence map indicates that whilst there is qualitative systematic review evidence focusing on the barriers and facilitators to the deprescribing of drugs that may cause dependency and antidepressants, little evidence examined views of specific interventions intended to promote adherence or aid deprescribing. In addition, gabapentinoids were not represented by any of the systematic reviews eligible for inclusion in the map.

Areas of the patient care pathway where groups of systematic review research with similar aims and medication types already exist. Twenty-two systematic reviews synthesised evaluations of interventions to promote the deprescribing of various drugs that may cause dependency and antidepressant medications, although these were mainly of low overall quality. In addition, all drugs that may cause dependency and antidepressant medication, aside from gabapentinoids, were included in at least one review focusing on evaluating interventions to optimise prescribing, although the quality of this evidence varied.

For research commissioners, the evidence and gap map indicates several areas where gaps in systematic review evidence have been identified and may benefit from searches for primary evidence in this area, depending on the nature of the future policy needs. Examples of these areas include qualitative evidence regarding patient and/or prescriber experiences of specific interventions to aid deprescribing or practitioner experiences of prescribing hypnotic medication. Systematic reviews on the optimisation of gabapentinoids are also absent from this map and should be a priority when considering commissioning of future systematic reviews. There is also an underrepresentation of systematic review evidence pertaining to shared decision-making within the map. The absence of systematic reviews, and abundance of poor-quality systematic reviews in others, means there is a lack of evidence to inform decision-making for policy makers in these areas. To address this, future systematic reviews to address these gaps should be conducted according to best-practice guidelines.^16^

The evidence and gap map may also be a useful resource for individuals involved in the prescription of drugs that may cause dependency and antidepressants, to locate evidence regarding the effectiveness of certain interventions to promote the taking of medication in line with patient needs or to support deprescribing where appropriate.

## Limitations

There are two main limitations with our study. First, many of the systematic reviews included in the evidence and gap map did not directly align with our research questions and many included medications outside the scope of our map. This may reduce the overall relevance of these systematic reviews to the evidence and gap map.

Second, the search strategy only sought systematic review evidence, thus, there may be primary evidence on this topic which may be useful for decision makers that has not been identified within this resource, as well as further systematic review evidence published since the searches for this review were conducted in August 2020. In addition, our search strategy did not seek systematic reviews that inform the development of NICE guidelines, which, whilst potentially relevant to the aims of this evidence and gap map, were not readily identified through bibliographic database searches.

## Conclusions

This evidence and gap map highlights the quantity, quality and range of systematic review evidence available to inform the optimal prescribing of drugs that may cause dependency and antidepressant medication. The map identifies areas where further evidence synthesis may be beneficial. Systematic reviews focusing on evaluating interventions to promote deprescribing would be particularly beneficial, as would reviews focusing on addressing the paucity of evidence regarding the deprescription of gabapentinoids. These reviews should be conducted with reference to best-practice guidelines to ensure their quality is sufficient to promote confidence in their findings for policy makers, commissioners and clinical practitioners. The evidence included within the evidence and gap map may support policy makers and service commissioners to develop appropriate support for individuals receiving a prescription of one or more of the medications included in this review. The evidence and gap may be used to inform the commissioning of further research and may also help inform the prescribing decisions of clinicians made alongside their patients.

## Supplemental Material

Supplemental Material - Optimising the prescribing of drugs that may cause dependency: An evidence and gap map of systematic reviews Click here for additional data file. Supplemental Material for Optimising the prescribing of drugs that may cause dependency: An evidence and gap map of systematic reviews by Liz Shaw, Michael Nunns, Simon Briscoe, Ruth Garside, Malcolm Turner, GJ Melendez-Torres, Hassanat M Lawal and Jo Thompson Coon in Journal of Health Services Research & Policy
