# Supplementary material for: Optimising the prescribing of drugs that may cause dependency: An evidence and gap map of systematic reviews
Source: J Health Serv Res Policy. 2023 May 29;28(4):271–81. doi: 10.1177/13558196231164592 (PMC10515472; doi:10.1177/13558196231164592)
Supplement: Supplemental Material - Optimising the prescribing of drugs that may cause dependency: An evidence and gap map of systematic reviews [file sj-pdf-1-hsr-10.1177_13558196231164592.pdf]

*Journal of Health Services Research & Policy*

**Optimising the prescribing of drugs that may cause dependency: an evidence and gap map of systematic reviews**

Shaw L, et al

# Online Supplement

## Evidence and gap map can be accessed here:

https://eppi.ioe.ac.uk/CMS/Portals/35/EGM%20University%20of%20Exeter%20August%202021.html

Under Review

PRISMA diagram

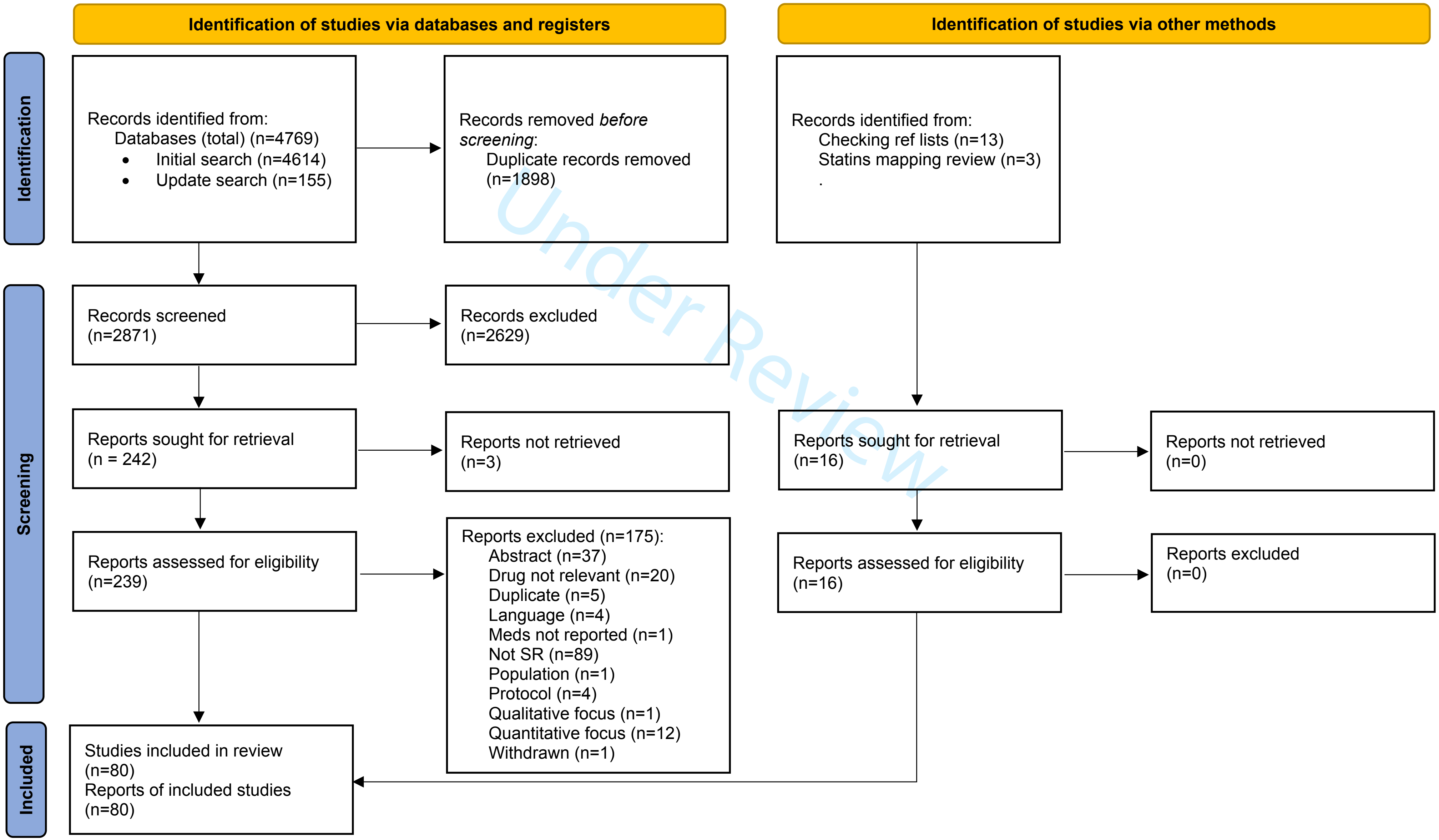


Under Review

*Journal of Health Services Research & Policy*
**Optimising the prescribing of drugs that may cause dependency: an evidence and gap map of systematic reviews**
Shaw L, et al

## Example search strategy and list of websites

Database: MEDLINE

Host: Ovid

Issue: 1946 to August 10, 2020

Date Searched: 11/8/2020

Searcher: SB

Hits: 875

Strategy:

1. ((appropriate* or discontinu* or enhance* or inappropriate* or incorrect* or "in correct*" or optim* or safe or suboptim* or "sub optim*" or tapering or withdrawal) adj4 (drug* or medicine* or medication* or prescri*)).tw.
2. Inappropriate Prescribing/
3. exp *Drug Prescriptions/
4. ((drug* or guideline* or guidance or medicine* or medication* or patient* or prescri*) adj4 (adhere* or compliance or concordance)).tw.
5. exp *Patient Compliance/
6. ((shared or sharing or informed) adj2 (decision* or choice*)).tw.

Under Review

*Journal of Health Services Research & Policy*
**Optimising the prescribing of drugs that may cause dependency: an evidence and gap map of systematic reviews**
Shaw L, et al

7. (decision adj2 (aid* or support*)).tw.
8. *Decision Making/
9. *decision support techniques/
10. ((consumer* or patient*) adj3 (involv* or participat*)).tw.
11. *patient participation/
12. ("patient cent*" adj2 (approach* or care or decision* or intervention* or treatment*)).tw.
13. Patient-Centered Care/
14. (behavi* adj2 chang*).tw.
15. (restriction adj2 (policy or policies)).tw.
16. or/1-15
17. benzodiazepine*.tw.
18. (alprazolam or flunitrazepam or chlordiazepoxide or clobazam or clonazepam or diazepam or lorazepam or midazolam or nitrazepam or oxazepam or prazepam or temazepam).tw.
19. exp Benzodiazepines/

Under Review

*Journal of Health Services Research & Policy*
**Optimising the prescribing of drugs that may cause dependency: an evidence and gap map of systematic reviews**
Shaw L, et al

20. (antidepres* or "anti depres*").tw.

21. (serotonin or norepinephrine or noradrenaline or neurotransmitter* or dopamin* or SSRI* or SNRI* or NARI* or SARI* or NDRI* or tricyclic* or tetracyclic*).tw.

22. exp Antidepressive Agents/

23. (opioid* or opiate*).tw.

24. (morphine or hydromorphone or levorphanol or meperidine or methadone or propoxyphene of codeine or pentazocine or hydrocodone or oxycodone or fentanyl or tramadol).tw.

25. exp Analgesics, Opioid/

26. "z drug*".tw.

27. (zopiclone or zolpidem or zaleplon or eszopiclone).tw.

28. exp "hypnotics and sedatives"/

29. (gabapentin* or mirogabalin or phenibut or pregabalin).tw.

30. Gabapentin/

31. or/17-30

32. 16 and 31

Under Review

33. statin*.tw.
34. ("HMG-CoA reductase inhibitor*" or "3-hydroxy-3-methyl-glutaryl-CoA reductase inhibitor*" or "3-hydroxy-3-methylglutaryl coenzyme A reductase inhibitor*" or "HMGCR inhibitor*" or "Hydroxymethylglutaryl-CoA Reductase Inhibitor*").tw.
35. exp Hydroxymethylglutaryl-CoA Reductase Inhibitors/
36. (atorvastatin or Lipitor or Torvast).tw.
37. (fluvastatin or Lescol).tw.
38. (lovastatin or Mevacor or Altocor or Altoprev).tw.
39. (pitavastatin or Livalo or Pitava).tw.
40. (pravastatin or Pravachol or Selektine or Lipostat).tw.
41. (rosuvastatin or Crestor).tw.
42. (simvastatin or Zocor or Lipex).tw.
43. antihypertensive*.tw.
44. exp Antihypertensive Agents/
45. ("ACE inhibitor*" or antagonist or "angiotensin II receptor" or "beta blocker*" or "calcium channel blocker*" or "thiazide diuretic*").tw.

Under Review

*Journal of Health Services Research & Policy*
**Optimising the prescribing of drugs that may cause dependency: an evidence and gap map of systematic reviews**
Shaw L, et al

46. (acebutolol or adrenomedullin or alprenolol or amlodipine or atenolol or bendroflumethiazide or bepridil or betaxolol or bethanidine or bimatoprost or bisoprolol or bosentan or "bretylium tosylate" or brimonidine tartrate or bupranolol or captopril or carteolol or carvedilol or celiprolol or chlorisondamine or chlorothiazide or chlorthalidone or cilazapril or clonidine or cromakalim or cyclopenthiazide or debrisoquin or diazoxide or dihydralazine or dihydroalprenolol or diltiazem or doxazosin or enalapril or enalaprilat or eplerenone or epoprostenol or felodipine or fenoldopam or fosinopril or guanabenz or guanethidine or guanfacine or hexamethonium or "hexamethonium compound*" or hydralazine or hydrochlorothiazide or hydroflumethiazide or indapamide or indoramin or irbesartan or isradipine or kallidin or ketanserin or labetalol or latanoprost or lisinopril or losartan or mecamylamine or methyldopa or metipranolol or metolazone or metoprolol or mibefradil or minoxidil or muzolimine or nadolol or nebivolol or nicardipine or nicorandil or nimodipine or nisoldipine or nitrendipine or nitroprusside or olmesartan medoxomil or oxprenolol or pargyline or pempidine or penbutolol or "pentolinium tartrate" or perindopril or phenoxybenzamine or phentolamine or pinacidil or pindolol or piperoxan or polythiazide or prazosin or propranolol or protoveratrines or quinapril or ramipril or reserpine or rilmenidine or telmisartan or teprotide or terlipressin or ticrynafen or timolol or todralazine or tolazoline or torsemide or travoprost or trichlormethiazide or trimethaphan or valsartan or "veratrum alkaloid*" or vincamine or xipamide).tw.

47. or/33-46

48. cardiovascular.tw.

49. exp Cardiovascular Diseases/

50. ((cardiac or coronary or heart) adj3 (arrest* or attack* or disease* or failure*)).tw.

51. ((heart or myocard* or transient) adj3 (infarc* or ischaemi* or ischemi*)).tw.

Under Review

*Journal of Health Services Research & Policy*
**Optimising the prescribing of drugs that may cause dependency: an evidence and gap map of systematic reviews**
Shaw L, et al

52. angina*.tw.
53. Angina Pectoris/
54. stroke*.tw.
55. exp Stroke/
56. or/48-55
57. 16 and 47 and 56
58. ((effectiveness or implementation or literature or map or mapping or qualitative or rapid or realist or systematic or scoping or "state of the art" or umbrella) adj2 (assessment* or overview* or review* or synthes*)).tw.
59. ("meta analy*" or metaanaly* or metasynthe* or "meta synthe*").tw.
60. ((systematic or evidence) adj1 assess*).tw.
61. (qualitative adj2 (evidence or synthes*)).tw.
62. (overarching adj2 model).tw.
63. "review* of reviews".tw.
64. systematic review.pt.

Under Review

65. meta-analysis.pt.

66. or/58-65

67. 32 and 66 [Drugs that cause dependency search results]

68. 57 and 66 [Statins search results]

Notes: Date limited 2010 to date of search.

Under Review

Websites:

Website: Royal Pharmaceutical Society Library Catalogue

URL: https://rpsgb.koha-ptfs.co.uk/

Date Searched: 21/10/2020

Searcher: SB

Hits: 73 hits

Strategy:

*Journal of Health Services Research & Policy*
**Optimising the prescribing of drugs that may cause dependency: an evidence and gap map of systematic reviews**
Shaw L, et al

Advanced search setting.

Keyword: opioid* OR

Keyword: benzodiazepine* OR

Keyword: antidepressant* OR

Keyword: gabapentinoid*

Website: Royal College of Physicians Library Catalogue

URL: https://rcp.soutron.net/Portal/Default/en-GB/Search/SimpleSearch

Date Searched: 21/10/2020

Searcher: SB

Hits: 6

Strategy:

All fields: opioid OR opioids OR benzodiazepine OR benzodiazepines OR antidepressant OR antidepressants OR gabapentinoid OR gabapentinoids

Under Review

*Journal of Health Services Research & Policy*
**Optimising the prescribing of drugs that may cause dependency: an evidence and gap map of systematic reviews**
Shaw L, et al

Website: Royal College of Psychiatrists Library Catalogue

URL: https://rcpsych.soutron.net/Portal/Default/en-GB/Search/SimpleSearch

Date Searched: 21/10/2020

Searcher: SB

Hits: 20

Strategy:

opioid* OR benzodiazepine* OR antidepressant* OR gabapentinoid*

Under Review

Website: Mind

URL: https://www.mind.org.uk/

Date Searched: 21/10/2020

Searcher: SB

Hits: 0

Strategy: Browsed website for relevant studies

*Journal of Health Services Research & Policy*
**Optimising the prescribing of drugs that may cause dependency: an evidence and gap map of systematic reviews**
Shaw L, et al

Website: Mental Health UK

URL: https://mentalhealth-uk.org/

Date Searched: 21/10/2020

Searcher: SB

Hits: 0

Strategy: Browsed website for relevant studies

Website: Priory Group

URL: https://www.priorygroup.com/

Date Searched: 21/10/2020

Searcher: SB

Hits: 0

Strategy: Browsed website for relevant studies

Under Review

*Journal of Health Services Research & Policy*

**Optimising the prescribing of drugs that may cause dependency: an evidence and gap map of systematic reviews**

Shaw L, et al

# Details on stakeholder consultation

## Clinical stakeholder and patient and public involvement (PPI)

Different groups of stakeholders were provided the opportunity to control or influence the systematic mapping review at key points during the project's development. Clinical and research stakeholders were recruited to the project via our existing relationships with NHSE-I and the University of Exeter. Patients with lived experience were recruited via the existing PenARC Patient Engagement group.

Four teleconferences were conducted with different clinical and research stakeholder groups during the project. In the first meeting, stakeholders from NICE, HEE, University of Birmingham, and NHSE-I supported the team to develop the bibliographic database search strategies and finalise the protocol. Their feedback directly shaped the direction of the project at this stage. The following three teleconferences provided the opportunity for these stakeholders to influence the project by focused on issues relating to developing the patient care pathway or the draft evidence and gap map and dissemination of the findings. These meetings were supplemented with email contact when we had specific questions relevant to the protocol, screening and data extraction stages of the project.

Regarding involvement of the PPI group three 60-90 min meetings were held between June and December 2020. Each meeting was held via Zoom and was attended by at least one member of the research team (KL, LS, MT or SB) and 3-5 people with experience of taking one or more of the medications of interest to this review. Feedback from the first PPI meeting enabled us to refine our research questions and inclusion criteria. In the second meeting, conducted during development of the patient care pathways, we consulted with patients and carers with experience of taking one or more of these medications to check to what extent the pathways reflected their experience of accessing, being prescribed, adhering to and discontinuing their medication. Care pathways were amended in light of the comments received. We also asked clinicians with relevant topic expertise to comment on how well each pathway reflected their experience and knowledge of working within the area. Both groups were

Under Review

given the opportunity to comment on the level of detail provided in the final evidence and gap map, and its presentation to ensure the findings were accessible to multiple audiences.

## Data extracted from each review

Under Review

Data extracted included:

- Bibliographic information
- Review focus/aim
- Type of review
- Type of studies included
- Type of synthesis
- Type of medication being prescribed
- Medical condition(s) being treated

*Journal of Health Services Research & Policy*
**Optimising the prescribing of drugs that may cause dependency: an evidence and gap map of systematic reviews**
Shaw L, et al

- Age at which study participants eligible for inclusion (Deviation from protocol which stated: Mean age of population)
- If review inclusion criteria and/or synthesis plan considered ethnicity of sample (Deviation from protocol which stated: Ethnicity of sample)
- If review inclusion criteria and/or synthesis plan considered socio-economic status of sample (Deviation from protocol which stated: Socio-economic status of sample)
- Intervention name and aim (Protocol only specified intervention name)
- Perspectives obtained (Qualitative studies only)
- Experiences of which phenomenon (Qualitative studies only – additional information to that specified in protocol)
- Service/part of care pathway (See Development of patient care pathways for further detail)
- Setting/Context (Additional to that specified in protocol)
- Outcomes evaluated

CEESAT criteria:

Under Review

*Journal of Health Services Research & Policy*
**Optimising the prescribing of drugs that may cause dependency: an evidence and gap map of systematic reviews**
Shaw L, et al

In order to identify the highest quality systematic reviews for inclusion in the evidence and gap map, two reviewers (LS, MN) independently applied four criteria from the Collaboration for Environmental Evidence Synthesis Assessment Tool (CEESAT) to each study eligible for inclusion in the review.[24] These criteria were as follows:

1. Search strategy: Is approach to searching clearly defined, systematic and transparent?
2. Is search comprehensive?
3. Does the review critically appraise each study?
4. During critical appraisal was an effort made to minimise subjectivity?

The reviewers identified items where each of the included reviews did not score at least a "Green", with disagreements on any item resolved through discussion. The standards required to score a "Green" on each criteria are outlined in Table 3 below. A score lower than 'Green' indicates poor quality or absence of reported information.

*Table 3: Criteria required to score "Green" Rating on CEESAT items*

| **CEESAT criteria** | **Standard necessary to achieve Green rating** |
|---|---|

Under Review

*Journal of Health Services Research & Policy*
**Optimising the prescribing of drugs that may cause dependency: an evidence and gap map of systematic reviews**
Shaw L, et al

| | |
|---|---|
| Search strategy: Is approach to searching clearly defined, systematic and transparent? | All search terms, Boolean operators ('AND', 'OR' etc.) and wildcards are clearly stated so that the exact search is repeatable by a third party<br>AND<br>There is information about the sources searched, together with dates of search [but no limitations justified (e.g. language, or publication date, no grey literature searches) |
| Is search comprehensive? | Original: Sources of articles searched capture both conventionally published scientific literature and grey literature using a combination of databases, search engines |

Under Review

*Journal of Health Services Research & Policy*
**Optimising the prescribing of drugs that may cause dependency: an evidence and gap map of systematic reviews**
Shaw L, et al

| | |
|---|---|
| | and specialist websites (may also be informed by stakeholders) or limitations are fully justified.<br><br>Modified: Require a minimum of 3 databases, AND at least one other source to avoid rating of amber. Specific searches for grey literature are NOT necessary |
| Does the review critically appraise each study? | Green: An effort is made to identify relevant sources of bias (threats to internal and<br>external validity)<br>AND<br>Each type of bias or threat to internal and external validity is assessed individually for |

Under Review

*Journal of Health Services Research & Policy*
**Optimising the prescribing of drugs that may cause dependency: an evidence and gap map of systematic reviews**
Shaw L, et al

| | |
|---|---|
| | all included studies and reported on a critical appraisal sheet. |
| During critical appraisal was an effort made to minimise subjectivity? | Original: An effort is made to minimise subjectivity by predefining critical appraisal process in a protocol<br>AND<br>At least two people critically appraised each study but not independently (e.g. second person aware of first person's decision) OR a subset of studies was appraised by at least two people independently and disagreements and process of resolution reported. |

Under Review

*Journal of Health Services Research & Policy*
**Optimising the prescribing of drugs that may cause dependency: an evidence and gap map of systematic reviews**
Shaw L, et al

| | |
|---|---|
| | Modified: do not need to check protocol; do NOT need mention of process for resolving disagreements to avoid Amber, AS LONG AS it is clearly stated that two reviewers performed appraisal independently |

Under Review

Modified AMSTAR-2 criteria

We adapted the AMSTAR-2 by adding items from the reporting standards for qualitative evidence synthesis to enhance the tools applicability to reviews of qualitative evidence. These items were as follows:

1. Is synthesis methodology and/or theoretical framework clearly described and justified?
2. Was the description and rationale for any sampling described?
3. Was the process of data extraction clearly described?

*Journal of Health Services Research & Policy*
**Optimising the prescribing of drugs that may cause dependency: an evidence and gap map of systematic reviews**
Shaw L, et al

4. Was the process for coding of data clearly described?
5. Was the process of developing themes clearly described?
6. Do the authors reflect on how their theoretical or ideological perspectives may have influenced their review findings?
7. Was use of any software clearly described?

Under Review

## Updated search results

Summary of studies (n=2) identified for inclusion following update searches conducted 2nd August 2022.

| | | | | | |
|---|---|---|---|---|---|
| **Reference** | Coronado-Vázquez V, Canet-Fajas C, Delgado-Marroquín MT, Magallón-Botaya R, Romero-Martín M, Gómez-Salgado J. Interventions to facilitate shared decision-making using decision aids with patients in Primary Health Care: A systematic review. Medicine (Baltimore). 2020 Aug 7;99(32):e21389. doi: 10.1097/MD.0000000000021389. | | | | |
| **Author** | **Year** | **Quality appraised using AMSTAR 2 (Y/N): QA score*** | **Topic [Type of evidence]** | **Medication of interest** | **Care pathway** |
| Coronado- Vázquez[s13] | 2020 | N: Critically-low | Evaluating intervention to optimise patient adherence to a medication [Quantitative] | Anti-depressants | Pre-treatment/ initiation, Maintaining treatment |

*based on CEESAT appraisal criteria. Note this review had 24 included studies, including 1 study focused on children.

| | |
|---|---|
| **Reference** | Xie CX, Chen Q, Hincapié CA, Hofstetter L, Maher CG, Machado GC. Effectiveness of clinical dashboards as audit and feedback or clinical decision support tools on medication use and test ordering: a systematic review of randomized controlled trials. J Am Med Inform Assoc. 2022 Sep 12;29(10):1773-1785. doi: 10.1093/jamia/ocac094. |

*Journal of Health Services Research & Policy*

**Optimising the prescribing of drugs that may cause dependency: an evidence and gap map of systematic reviews**

Shaw L, et al

| **Author** | **Year** | **Quality appraised using AMSTAR 2 (Y/N): QA score*** | **Topic [Type of evidence]** | **Medication of interest** | **Care pathway** |
|---|---|---|---|---|---|
| Xie[s78] | 2022 | N: Critically-low | Evaluating intervention to optimise prescribing [Quantitative] | Opioids | Pre-treatment/ initiation |

*based on CEESAT appraisal criteria

Under Review

*Table 1: Scores on CEESAT items - included studies*

| First Author, Date | Search strategy: Is approach to searching clearly defined, systematic and transparent? | Is search comprehensive? | Does the review critically appraise each study? | During critical appraisal was an effort made to minimise subjectivity? | Total Score |
|---|---|---|---|---|---|

*Journal of Health Services Research & Policy*

**Optimising the prescribing of drugs that may cause dependency: an evidence and gap map of systematic reviews**

Shaw L, et al

| | | | | | |
|---|---|---|---|---|---|
| Al Shemeili 2015 | Green | Green | Green | Green | 0 |
| Baandrup 2018 | Green | Green | Green | Green | 0 |
| Brown 2019 | Green | Green | Green | Green | 0 |
| Dills 2018 | Green | Green | Green | Green | 0 |
| Eccleston 2017 | Green | Green | Green | Green | 0 |
| Garcia-Perez 2011 | Green | Green | Green | Green | 0 |
| Gillaizeau 2013 | Green | Green | Green | Green | 0 |
| Gould 2014 | Green | Green | Green | Green | 0 |
| Hoyle 2018 | Green | Green | Green | Green | 0 |
| Hukins 2019 | Green | Green | Green | Green | 0 |
| Low 2015 | Green | Green | Green | Green | 0 |
| Lynch 2020 | Green | Green | Green | Green | 0 |
| Mathieson 2020 | Green | Green | Green | Green | 0 |
| Maund 2019a | Green | Green | Green | Green | 0 |
| Maund 2019b | Green | Green | Green | Green | 0 |
| Moise 2018 | Green | Green | Green | Green | 0 |
| Pantoja 2019 | Green | Green | Green | Green | 0 |
| Posadzki 2016 | Green | Green | Green | Green | 0 |
| Tedesco 2017 | Green | Green | Green | Green | 0 |
| Zhang 2020a | Green | Green | Green | Green | 0 |
| Zhang 2020b | Green | Green | Green | Green | 0 |

*Journal of Health Services Research & Policy*
**Optimising the prescribing of drugs that may cause dependency: an evidence and gap map of systematic reviews**
Shaw L, et al

| | | | | | |
|---|---|---|---|---|---|
| Nieuwlaat 2014 | Green | Green | Green | Green | 0 |
| Al-Jumah 2012 | Amber | Green | Green | Green | 1 |
| Awadalla 2020 | Green | Amber | Green | Green | 1 |
| Beaudion 2016 | Green | Green | Amber | Green | 1 |
| Becker 2016 | Amber | Green | Green | Green | 1 |
| Dagenais 2010 | Green | Amber | Green | Green | 1 |
| Darker 2015 | Green | Green | Green | Red | 1 |
| Ernstzen 2017 | Amber | Green | Green | Green | 1 |
| Frank 2017 | Green | Green | Amber | Green | 1 |
| Garcia-Perez 2020 | Green | Amber | Green | Green | 1 |
| Herzig 2018 | Amber | Green? | Green | Green | 1 |
| Hopkins 2019 | Green | Green | Amber | Green | 1 |
| Huang 2013 | Green | Amber | Green | Green | 1 |
| Kimmel 2019 | Green | Green | Green | Red | 1 |
| King 2018[42] | Green | Green | Amber | Green | 1 |
| Milosavljevic 2018 | Green | Green | Amber | Green | 1 |
| Mugunthan 2011 | Green | Amber | Green | Green | 1 |
| Mokhar 2018 | Green | Amber | Green | Green | 1 |
| Morera-Fumero 2020 | Green | Amber | Green | Green | 1 |
| Nuckols 2014 | Amber | Green | Green | Green | 1 |
| Nussbaumer 2014 | Green | Green | Amber | Green | 1 |
| Ostini 2011 | Green | Green | Amber | Green | 1 |
| Page 2016 | Green | Green | Amber | Green | 1 |
| Park 2017 | Green | Green | Amber | Green | 1 |

*Journal of Health Services Research & Policy*
**Optimising the prescribing of drugs that may cause dependency: an evidence and gap map of systematic reviews**
Shaw L, et al

| | | | | | |
|---|---|---|---|---|---|
| Readdean 2018 | Green | Amber | Green | Green | 1 |
| Reeve 2017 | Green | Green | Green | Red | 1 |
| Rubio-Valera 2011 | Green | Green | Amber | Green | 1 |
| Sirdifield 2013 | Green | Green | Amber | Green | 1 |
| Wilson 2019 | Green | Green | Green | Red | 1 |
| Wong 2020 | Green | Green | Amber | Green | 1 |
| Conn 2016 | Amber | Green | Green | Green | 1 |
| Bourcier 2017 | Green | Amber | Amber | Green | 2 |
| Dann-Reed 2020 | Green | Green | Amber | Amber | 2 |
| Loganathan 2011 | Green | Amber | Amber | Green | 2 |
| Anderson 2014 | Green | Amber | Green | Amber | 2 |
| Cullinan 2013 | Green | Amber | Amber | Green | 2 |
| Furlan 2018 | Green | Green | Amber | Amber | 2 |
| Griffiths 2012 | Green | Green | Amber | Red | 2 |
| Hudson 2019 | Green | Green | Amber | Red | 2 |
| Kennedy 2019 | Green | Amber | Amber | Green | 2 |
| Kunstler 2019 | Green | Green | Amber | Red | 2 |
| Mauri 2020 | Green | Green | Amber | Red | 2 |
| Nguyen 2020 | Green | Amber | Amber | Green | 2 |
| Rashid 2018 | Green | Amber | Amber | Green | 2 |
| Reeve 2013 | Green | Green | Amber | Red | 2 |
| Sirdifield 2017 | Green | Amber | Amber | Green | 2 |
| Toye 2017 | Green | Amber | Amber | Green | 2 |
| Weatherburn 2020 | Green | Amber | Green | Red | 2 |

*Journal of Health Services Research & Policy*
**Optimising the prescribing of drugs that may cause dependency: an evidence and gap map of systematic reviews**
Shaw L, et al

| | | | | | |
|---|---|---|---|---|---|
| Wetzel 2018 | Green | Amber | Amber | Green | 2 |
| Wilbur 2018 | Amber | Green | Amber | Green | 2 |
| Chong 2011 | Green | Amber | Amber | Red | 3 |
| Ford 2017 | Amber | Green | Amber | Red | 3 |
| Hart 2020 | Amber | Amber | Amber | Green | 3 |
| Mayer 2010 | Amber | Green | Amber | Red | 3 |
| Polinski 2011 | Amber | Amber | Amber | Green | 3 |
| Pollman 2015 | Amber | Green | Amber | Red | 3 |
| Chhina 2013 | Amber | Amber | Amber | Red | 4 |

nder Review

*Journal of Health Services Research & Policy*
**Optimising the prescribing of drugs that may cause dependency: an evidence and gap map of systematic reviews**
Shaw L, et al

*Table 2: Scores on AMSTAR-2 tool for studies with CEESAT score of 0*

| Study (First author, date) | 1. PICO components | 2. Protocol | 3. Study design explanation | 4. Comprehensive search strategy | 5. Duplicate study selection | 6. Duplicate data extraction | 7. Details of excluded studies | 8. Description of included studies | 9b. RoB assessment (NRSIs) | 10. Funding sources | 9a. Risk of Bias (RoB) assessment (RCTs) | 11a. RCTs Meta-analysis | 11b. NRSIs Meta-analysis (MA) | 12. MA: RoB in individual studies | 13. Qualitative reviews: Is synthesis methodology /theoretical framework clearly described /justified? | 14. Qualitative reviews: was description /rationale for sampling described? | 15. Qualitative reviews: was process of data extraction clearly described? | 16. Qualitative reviews: was process for coding data clearly described? | 17. Qualitative reviews: was process of developing themes clearly described? | 18. Qualitative reviews: Do authors reflect on how their theoretical /ideological perspectives may influence their findings? | 19 Qualitative reviews: Was use of any software clearly described? | 20. RoB: discussion of results | 21. Heterogeneity | 22. Publication bias | 23. Reports conflicts of interest | Overall rating |
|---|---|---|---|---|---|---|---|---|---|---|---|---|---|---|---|---|---|---|---|---|---|---|---|---|---|---|
| Al Shemeili, 2015 | Y | Y | Y | Y | Y | Y | Y | Y | Y | N | NA | NA | N | NA | NA | NA | NA | NA | NA | NA | NA | Y | Y | NA | N | H |
| Baandrup 2018 | Y | Y | N | Y | Y | Y | Y | Y | NA | Y | Y | Y | NA | Y | NA | NA | NA | NA | NA | NA | NA | Y | Y | Y | Y | H |
| Brown 2019 | Y | Y | N | Y | Y | Y | Y | Y | NA | Y | Y | Y | NA | Y | NA | NA | NA | NA | NA | NA | NA | Y | Y | Y | Y | H |
| Dills 2018 | Y | N | N | Y | Y | Y | N | Y | NA | Y | Y | N | NA | NA | NA | NA | NA | NA | NA | NA | NA | Y | Y | NA | Y | L |
| Eccleston 2017 | Y | Y | N | Y | Y | Y | Y | Y | NA | Y | Y | N | NA | NA | NA | NA | NA | NA | NA | NA | NA | Y | Y | NA | Y | H |
| Garcia-Perez 2011 | Y | N | N | Y | Y | Y | N | Y | NA | N | Y | N | NA | NA | NA | NA | NA | NA | NA | NA | NA | Y | Y | NA | N | L |
| Gillaizeau 2013 | Y | Y | Y | Y | Y | Y | Y | Y | Y | Y | Y | Y | Y | Y | NA | NA | NA | NA | NA | NA | NA | Y | Y | Y | Y | H |
| Gould 2014 | Y | N | o | Y | Y | Y | N | Y | NA | N | Y | Y | NA | Y | NA | NA | NA | NA | NA | NA | NA | Y | Y | Y | Y | M |
| Hoyle 2018 | Y | Y | N | Y | Y | Y | N | N | Y | N | Y | N | N | NA | NA | NA | NA | NA | NA | NA | NA | Y | Y | NA | Y | M |
| Hukins 2019 | Y | N | N | Y | Y | Y | N | Y | Y | N | NA | NA | N | NA | NA | NA | NA | NA | NA | NA | NA | Y | Y | NA | Y | L |

*Journal of Health Services Research & Policy*
**Optimising the prescribing of drugs that may cause dependency: an evidence and gap map of systematic reviews**
Shaw L, et al

| Study (First author, date) | 1. PICO components | 2. Protocol | 3. Study design explanation | 4. Comprehensive search strategy | 5. Duplicate study selection | 6. Duplicate data extraction | 7. Details of excluded studies | 8. Description of included studies | 9b. RoB assessment (NRSIs) | 10. Funding sources | 9a. Risk of Bias (RoB) assessment (RCTs) | 11a. RCTs Meta-analysis | 11b. NRSIs Meta-analysis (MA) | 12. MA: RoB in individual studies | 13. Qualitative reviews: Is synthesis methodology /theoretical framework clearly described /justified? | 14. Qualitative reviews: was description /rationale for sampling described? | 15. Qualitative reviews: was process of data extraction clearly described? | 16. Qualitative reviews: was process for coding data clearly described? | 17. Qualitative reviews: was process of developing themes clearly described? | 18. Qualitative reviews: Do authors reflect on how their theoretical /ideological perspectives may influence their findings? | 19 Qualitative reviews: Was use of any software clearly described? | 20. RoB: discussion of results | 21. Heterogeneity | 22. Publication bias | 23. Reports conflicts of interest | Overall rating |
|---|---|---|---|---|---|---|---|---|---|---|---|---|---|---|---|---|---|---|---|---|---|---|---|---|---|---|
| Low 2015 | N | N | N | Y | Y | Y | N | Y | Y | N | Y | N | N | NA | NA | NA | NA | NA | NA | NA | NA | Y | Y | NA | Y | M |
| Lynch 2020 | Y | N | N | Y | Y | Y | N | PY | NA | N | Y | Y | NA | Y | NA | NA | NA | NA | NA | NA | NA | Y | Y | N | Y | L |
| Mathieson 2020 | Y | Y | N | Y | Y | Y | N | Y | NA | Y | Y | N | NA | NA | NA | NA | NA | NA | NA | NA | NA | Y | Y | NA | Y | M |
| Maund 2019a | Y | Y | NA | Y | Y | Y | N | Y | Y | N | NA | NA | N | NA | PY | Y | Y | Y | Y | N | Y | Y | Y | NA | Y | M |
| Maund 2019b | Y | Y | N | Y | Y | Y | Y | Y | Y | Y | Y | Y | Y | No | NA | NA | NA | NA | NA | NA | NA | N | N | N | Y | CL |
| Moise 2018 | Y | Y | N | Y | Y | Y | N | Y | Y | N | Y | N | N | NA | NA | NA | NA | NA | NA | NA | NA | Y | Y | NA | Y | M |
| Nieuwlaat 2014 | Y | Y | N | Y | Y | Y | Y | Y | NA | N | Y | N | NA | NA | NA | NA | NA | NA | NA | NA | NA | Y | Y | NA | Y | M |
| Pantoja 2019 | Y | Y | N | Y | Y | Y | Y | Y | Y | Y | Y | N | N | NA | NA | NA | NA | NA | NA | NA | NA | Y | Y | NA | Y | H |
| Posadzki 2016 | Y | Y | Y | Y | Y | Y | Y | Y | Y | Y | Y | Y | Y | Y | NA | NA | NA | NA | NA | NA | NA | Y | Y | N | Y | H |
| Tedesco 2017 | Y | Y | Y | Y | Y | Y | N | PY | NA | Y | Y | Y | NA | Y | NA | NA | NA | NA | NA | NA | NA | Y | Y | Y | Y | M |

*Journal of Health Services Research & Policy*
**Optimising the prescribing of drugs that may cause dependency: an evidence and gap map of systematic reviews**
Shaw L, et al

| Study (First author, date) | 1. PICO components | 2. Protocol | 3. Study design explanation | 4. Comprehensive search strategy | 5. Duplicate study selection | 6. Duplicate data extraction | 7. Details of excluded studies | 8. Description of included studies | 9b. RoB assessment (NRSIs) | 10. Funding sources | 9a. Risk of Bias (RoB) assessment (RCTs) | 11a. RCTs Meta-analysis | 11b. NRSIs Meta-analysis (MA) | 12. MA: RoB in individual studies | 13. Qualitative reviews: Is synthesis methodology /theoretical framework clearly described /justified? | 14. Qualitative reviews: was description /rationale for sampling described? | 15. Qualitative reviews: was process of data extraction clearly described? | 16. Qualitative reviews: was process for coding data clearly described? | 17. Qualitative reviews: was process of developing themes clearly described? | 18. Qualitative reviews: Do authors reflect on how their theoretical /ideological perspectives may influence their findings? | 19 Qualitative reviews: Was use of any software clearly described? | 20. RoB: discussion of results | 21. Heterogeneity | 22. Publication bias | 23. Reports conflicts of interest | Overall rating |
|---|---|---|---|---|---|---|---|---|---|---|---|---|---|---|---|---|---|---|---|---|---|---|---|---|---|---|
| Zhang 2020a | Y | Y | Y | Y | Y | Y | N | Y | Y | Y | NA | NA | N | NA | NA | NA | NA | NA | NA | NA | NA | Y | Y | NA | Y | H |
| Zhang 2020b | N | Y | N | Y | Y | Y | N | N | Y | N | Y | N | N | NA | NA | NA | NA | NA | NA | NA | NA | Y | Y | NA | Y | M |

CL=Critically Low overall quality; H=High overall quality; Low overall quality; M=Moderate overall quality; N=No; NA=Not Applicable; NRSI=Non-Randomised Studies of healthcare Interventions; PICO=Population, Intervention, Comparator, Outcome; PY=Partial Yes; RCT=Randomised Controlled Trial; RoB=Risk of Bias; Y=Yes cable, NR=Not Reported, NSAID=Non-Steroidal Anti-Inflammatories

*Journal of Health Services Research & Policy*
**Optimising the prescribing of drugs that may cause dependency: an evidence and gap map of systematic reviews**
Shaw L, et al

*Table 3: Review Overview*

Key for interpreting tables

| | |
|---|---|
| Green highlighted text: | High overall quality as appraised by AMSTAR-2 |
| Turquoise highlighted text: | Medium overall quality as appraised by AMSTAR-2 |
| Orange highlighted text: | Low overall quality as appraised by AMSTAR-2 |
| Red highlighted text: | Critically-low quality as appraised by the AMSTAR-2 |
| CEESAT Set 1: | Prioritised for full quality appraisal using AMSTAR-2 |
| CEESAT Set 2: | Scored poorly on 1 out of 4 items on CEESAT |
| CEESAT Set 3: | Scored poorly on 2-4 items on CEESAT |

Reviews were judged to be of 'High' relevance if review focused on evaluating intervention/gathering experiences regarding medication of interest within health/social care services relevant to UK service setting, 'Medium' relevance: if review aim/inclusion criteria somewhat relevant to aim of systematic mapping review, but also includes other medications not of interest, or quantity of relevant information is limited, 'Low' relevance: if quantity of relevant information low and/or intervention being evaluated not relevant to UK health/social care setting

Under Review

*Journal of Health Services Research & Policy*
**Optimising the prescribing of drugs that may cause dependency: an evidence and gap map of systematic reviews**
Shaw L, et al

| **Focus/Aim** | **Study (First author, date)** | **Type of Review (design of studies included), synthesis methods** | **Eligible age of participants within studies included in the review** | **Medications of interest** | **Medical condition for which medication of interest being taken** | **Relevance of systematic review to aim of evidence and gap map*** | **CEESAT set** | **Other comments** |
|---|---|---|---|---|---|---|---|---|
| Evaluating intervention: deprescribing | Al Shemeili 2015 | SR (Quantitative: prospective/ retrospective cohort, case control, cross-sectional), Narrative | 65 years or over | Hypnotics/Z-drugs | NR | Medium | 1 | |
| Evaluating intervention: enhance adherence | Al-Jumah 2012 | SR (Quantitative: RCTs), Narrative | 18 years or over<br>*Adult patients* | Antidepressants | Depression | High | 2 | |
| Practitioner views: reducing inappropriate prescribing | Anderson 2014 | SR (Qualitative: original research articles with qualitative component (i.e., qualitative, mixed or multimethod: survey, focus groups, semi structured interviews), Thematic | NR<br>*Included studies had a focus of: all ages (n=8), 'older patients' (n=13)* | Benzodiazepines<br>Opiates<br>Antidepressants<br>Hypnotics/Z-drugs | Depression<br>NR | Medium | 3 | |
| Evaluating intervention: optimise prescribing | Awadalla 2020 | SR (Quantitative: RCTs, pre-post intervention, cohort, case-control), Narrative | NR<br>*Where reported, description of included studies indicated included participants 18 years or above* | Opiates | Other<br>*Acute pain, post-operative pain* | High | 2 | |

Under review

*Journal of Health Services Research & Policy*
**Optimising the prescribing of drugs that may cause dependency: an evidence and gap map of systematic reviews**
Shaw L, et al

| Focus/Aim | Study (First author, date) | Type of Review (design of studies included), synthesis methods | Eligible age of participants within studies included in the review | Medications of interest | Medical condition for which medication of interest being taken | Relevance of systematic review to aim of evidence and gap map* | CEESAT set | Other comments |
|---|---|---|---|---|---|---|---|---|
| Evaluating intervention: deprescribing | Baandrup 2018 | SR (Quantitative: RCTs), MA | 18 years or over | Benzodiazepines Hypnotics/Z-drugs | Anxiety | High | 1 | |
| Evaluating intervention: optimise prescribing Guidelines | Beaudoin 2016 | SR (Quantitative: RCTs, cohort, time series, cross sectional), Narrative | NR | Opiates | Chronic pain | High | 2 | |
| Evaluating intervention: optimise prescribing | Becker 2016 | SR (Quantitative: RCT, two- group observational point-of-service, observational point-of-service studies), Narrative | NR | Antidepressants | Depression | Low | 2 | Intervention not relevant to UK setting |
| Evaluating intervention: optimise prescribing Directed at practitioners and patients | Bourcier 2018 | SR (Quantitative: BA, multicentre RCTs), Narrative | Other *Adults (n=19); Elderly (n=12)* | Benzodiazepines Antidepressants Hypnotics/Z-drugs | Insomnia *Sleep disorders* | High | 3 | |

Under review

*Journal of Health Services Research & Policy*

**Optimising the prescribing of drugs that may cause dependency: an evidence and gap map of systematic reviews**

Shaw L, et al

| **Focus/Aim** | **Study (First author, date)** | **Type of Review (design of studies included), synthesis methods** | **Eligible age of participants within studies included in the review** | **Medications of interest** | **Medical condition for which medication of interest being taken** | **Relevance of systematic review to aim of evidence and gap map*** | **CEESAT set** | **Other comments** |
|---|---|---|---|---|---|---|---|---|
| Evaluating intervention: enhance adherence | Brown 2019 | SR (Quantitative: RCTs, cluster-RCTs), MA+<br>Narrative | 16 years or over | Antidepressants | Depression | High | 1 | |
| Evaluating intervention: optimise prescribing | Chhina 2013 | SR (Quantitative: RCT, observational studies with a control group), Narrative | NR<br>*N=1 focused on children, n=3 focused on elderly* | Benzodiazepines<br>Antidepressants | Insomnia<br>Depression<br>Anxiety | Medium | 3 | One included study focused on children |
| Evaluating intervention: enhance adherence | Chong 2011 | SR (Quantitative: RCT), Narrative | 18 years or over | Antidepressants | Depression | High | 3 | |
| Evaluating intervention: enhance adherence | Conn 2016 | SR (Quantitative: RCTs, non-RCTs, Pre-experimental before and after), Narrative+MA | NR<br>*Adults. Median mean age across included studies 53 years* | Antidepressants | Depression | Medium | 2 | Medications of interest inferred from population disease characteristic |
| Practitioner views: reducing inappropriate prescribing | Cullinan 2014 | SR (Qualitative: Grounded theory, semi-structured interviews, focus groups, observation), Meta-ethnography | 65 years or over | Benzodiazepines<br>Opiates<br>Antidepressants | Depression<br>NR<br>Chronic pain<br>Insomnia<br>Anxiety | Medium | 3 | |

*Journal of Health Services Research & Policy*
**Optimising the prescribing of drugs that may cause dependency: an evidence and gap map of systematic reviews**
Shaw L, et al

| **Focus/Aim** | **Study (First author, date)** | **Type of Review (design of studies included), synthesis methods** | **Eligible age of participants within studies included in the review** | **Medications of interest** | **Medical condition for which medication of interest being taken** | **Relevance of systematic review to aim of evidence and gap map*** | **CEESAT set** | **Other comments** |
|---|---|---|---|---|---|---|---|---|
| Guidelines<br>Evaluating intervention: optimise prescribing | Dagenais 2010 | SRN Guideline (CPGs), Narrative | NR | Opiates | Chronic pain<br>*Lower back pain* | Medium | 2 | |
| Evaluating intervention: enhance adherence<br>One aspect of broad aim<br>Evaluating intervention: deprescribing<br>One aspect of interventions | Dann-Reed 2020 | SR (Quantitative: RCTs, service evaluations, conference abstracts), Narrative | NR<br>*Interventions targeted at peiople affected by dementia* | Benzodiazepines | NR | Medium | 3 | Outcomes and interventions relevant to our review are included, but not clear focus of the review |
| Evaluating intervention: deprescribing | Darker 2015 | SR (Quantitative: RCTs), Narrative+<br>MA | 16 years or over | Benzodiazepines | Insomnia<br>Anxiety<br>Other<br>*Illicit use* | Medium | 2 | Inclusion of some studies where drug use is illicit |

Under review

*Journal of Health Services Research & Policy*
**Optimising the prescribing of drugs that may cause dependency: an evidence and gap map of systematic reviews**
Shaw L, et al

| **Focus/Aim** | **Study (First author, date)** | **Type of Review (design of studies included), synthesis methods** | **Eligible age of participants within studies included in the review** | **Medications of interest** | **Medical condition for which medication of interest being taken** | **Relevance of systematic review to aim of evidence and gap map*** | **CEESAT set** | **Other comments** |
|---|---|---|---|---|---|---|---|---|
| Evaluating intervention: deprescribing | Dills 2018 | SR (Quantitative: RCTs), Narrative | 18 years or over | Benzodiazepines<br>Hypnotics/Z-drugs<br>Antidepressants | Depression<br>Other Illnesses associated with use of: alendronate, PPI, H2 antagonist<br>Insomnia<br>Anxiety | Medium | 1 | Palliative care studies where life expectancy is > 6 months included |
| Evaluating intervention: deprescribing | Eccleston 2017 | SR (Quantitative: RCTs), Narrative | 18 years or over | Opiates | Chronic pain | High | 1 | |
| Guidelines<br>Evaluating intervention: optimise prescribing | Ernstzen 2017 | SR (CPGs), Narrative | NA | Opiates | Chronic pain<br>*Chronic muscular-skeletal pain* | High | 2 | |

Under review

*Journal of Health Services Research & Policy*

**Optimising the prescribing of drugs that may cause dependency: an evidence and gap map of systematic reviews**

Shaw L, et al

| **Focus/Aim** | **Study (First author, date)** | **Type of Review (design of studies included), synthesis methods** | **Eligible age of participants within studies included in the review** | **Medications of interest** | **Medical condition for which medication of interest being taken** | **Relevance of systematic review to aim of evidence and gap map*** | **CEESAT set** | **Other comments** |
|---|---|---|---|---|---|---|---|---|
| Practitioner views: prescribing medication of interest<br>Secondary aim/focus | Ford 2017 | SR (Qualitative: any results reported as text rather than numbers, including mixed-methods studies. Included qualitative methods: interviews, survey), Meta-ethnography | NR | Antidepressants | Depression<br>Anxiety<br>*to a lesser extent, but focus is on depression* | Medium | 3 | Only 5 included studies, and one theme relevant for inclusion |
| Evaluating intervention: deprescribing | Frank 2017 | SR (Quantitative: randomized trials, cohort studies, case–control studies, and case series), Narrative | 18 years or over | Opiates | Chronic pain | High | 2 | |
| Evaluating intervention: deprescribing<br>Evaluating intervention: optimise prescribing | Furlan 2018 | SR (Quantitative: Randomized, non-randomized), Narrative | NR | Opiates | Chronic pain<br>NR | Medium | 3 | Some studies include individuals receiving MMT or naloxone to prevent overdose.<br>Inclusion criteria include those taking opioids to relieve pain and those taking opioids to feel euphoria.<br>Plenty of information that isn't relevant to our RQs, focus on Canadian context |

Under review

*Journal of Health Services Research & Policy*

**Optimising the prescribing of drugs that may cause dependency: an evidence and gap map of systematic reviews**

Shaw L, et al

| Focus/Aim | Study (First author, date) | Type of Review (design of studies included), synthesis methods | Eligible age of participants within studies included in the review | Medications of interest | Medical condition for which medication of interest being taken | Relevance of systematic review to aim of evidence and gap map* | CEESAT set | Other comments |
|---|---|---|---|---|---|---|---|---|
| Evaluating intervention: enhance adherence | Garcia-Perez 2011 | SR (Other: cost-effectiveness review- Quantitative, Models or primary studies describing full economic evaluations), Narrative | 18 years or over *Excluded 'elderly people'* | Antidepressants | Depression | High | 1 | |
| Evaluating intervention: enhance adherence | Garcia-Perez 2020 | SR (Other cost-effectiveness- Quantitative primary or model-based studies that assessed both costs and effectiveness of at least two alternatives, i.e., full economic evaluations), Narrative | 18 years or over *Adult. Excluded 'elderly people'* | Antidepressants | Depression Anxiety Other *Psychosis, schizophrenia, affective disorders with psychotic features or schizoaffective disorder* | Medium | 2 | Includes other medications not of interest, adherence is secondary to cost-effectiveness outcomes |
| Evaluating intervention: optimise prescribing | Gillaizeau 2013 | SR (Quantitative: RCTs, non-RCTs, CBAs, ITS), Narrative+MA | NR *Healthcare professionals with responsibility for patient care* | Antidepressants | Depression HBP | Low | 1 | Includes other medications not of interest, only one study about antidepressants |

*Journal of Health Services Research & Policy*
**Optimising the prescribing of drugs that may cause dependency: an evidence and gap map of systematic reviews**
Shaw L, et al

| Focus/Aim | Study (First author, date) | Type of Review (design of studies included), synthesis methods | Eligible age of participants within studies included in the review | Medications of interest | Medical condition for which medication of interest being taken | Relevance of systematic review to aim of evidence and gap map* | CEESAT set | Other comments |
|---|---|---|---|---|---|---|---|---|
| Evaluating intervention: deprescribing | Gould 2014 | SR (Quantitative: RCTs), MA | 60 years or over<br>*Mean/median age 60 plus, minimum age of sample = 50* | Benzodiazepines | NR | High | 1 | |
| Evaluating intervention: optimise prescribing<br>Qualitative other | Griffiths 2012 | SR (Mixed: Qualitative and quantitative studies), methods of analysis unclear | Other<br>*Adults* | Benzodiazepines<br>Hypnotics/Z-drugs<br>Antidepressants | Insomnia<br>Depression<br>Anxiety | Low | 3 | Methods are uninformative, unclear of included study designs. Appears to use qualitative synthesis of both qualitative and quantitative studies. Focus on prison, range of drugs (although mainly relevant), findings don't necessarily fit into aims of evidence and gap map |
| Evaluating intervention: deprescribing<br>Secondary aim | Hart 2020 | SR (Quantitative: observational or intervention (e.g., RCTs and quasi-experimental design)), Narrative | Other<br>*60 or over* | Benzodiazepines<br>Opiates<br>Hypnotics/Z-drugs<br>Antidepressants | NR | Medium | 3 | Includes other medications not of interest to this review. Also approach reduction of drugs of interest in a round-about way, rather than directly wanting to reduce them |

*Journal of Health Services Research & Policy*
**Optimising the prescribing of drugs that may cause dependency: an evidence and gap map of systematic reviews**
Shaw L, et al

| **Focus/Aim** | **Study (First author, date)** | **Type of Review (design of studies included), synthesis methods** | **Eligible age of participants within studies included in the review** | **Medications of interest** | **Medical condition for which medication of interest being taken** | **Relevance of systematic review to aim of evidence and gap map*** | **CEESAT set** | **Other comments** |
|---|---|---|---|---|---|---|---|---|
| Guidelines<br>Evaluating intervention: optimise prescribing | Herzig 2018 | SR (Guidelines), Narrative | Other<br>*Adults* | Opiates | Other<br>*Acute, non-cancer pain* | High | 2 | |
| Evaluating intervention: optimise prescribing | Hopkins 2019 | SR (Quantitative: interventional studies (clinical trials and pre- and post-studies), Narrative | NR | Opiates | Post-operative pain<br>Other<br>*Acute pain/trauma pain* | High | 2 | |
| Evaluating intervention: deprescribing | Hoyle 2018 | SR (Quantitative: RCTs, non-RCTs, CTs, ITS, BA), Narrative | Other<br>*Nursing home setting* | Benzodiazepines | NR | Medium | 1 | |
| Evaluating intervention: enhance adherence<br>Secondary outcome/aim | Huang 2013 | SR (Quantitative: RCTs), MA | Other<br>*Any age. Where reported, mean age ranged from 57-70 years in included studies* | Antidepressants | Depression | Medium | 2 | Only 4 trials report adherence to antidepressants. Outcome not high priority in review |
| Evaluating intervention: enhance adherence | Hudson 2019 | SR (Quantitative: RCTs (individual or cluster randomized), MA and meta-regression | 18 years or over | Antidepressants | Depression<br>Anxiety | High | 3 | |

Under review

*Journal of Health Services Research & Policy*
**Optimising the prescribing of drugs that may cause dependency: an evidence and gap map of systematic reviews**
Shaw L, et al

| **Focus/Aim** | **Study (First author, date)** | **Type of Review (design of studies included), synthesis methods** | **Eligible age of participants within studies included in the review** | **Medications of interest** | **Medical condition for which medication of interest being taken** | **Relevance of systematic review to aim of evidence and gap map*** | **CEESAT set** | **Other comments** |
|---|---|---|---|---|---|---|---|---|
| Evaluating intervention: optimise prescribing | Hukins 2019 | SR (Quantitative: observational, non-randomized evaluation), Narrative | Other<br>*People with a diagnosis of/suspected dementia* | Benzodiazepines<br>Hypnotics/Z-drugs | NR | Medium | 1 | |
| Practitioner views: prescribing medication of interest | Kennedy 2019 | SR (Qualitative: Semi-structured interviews, recording of consultations with patients, focus groups), Thematic Network Analysis | NR | Opiates | Chronic pain | High | 3 | |
| Evaluating intervention: optimise prescribing<br>Secondary aim | Kimmel 2019 | SR (Quantitative: Any study design eligible. Included RCTs, observational), Narrative | 18 years or over | Opiates | Chronic pain<br>Other<br>*Cancer, non-cancer pain, opioid use disorder, substance use, psychiatric conditions, other chronic illnesses* | High | 2 | Includes some non-eligible population groups e.g. cancer pain, OUD/substance use |

*Journal of Health Services Research & Policy*
**Optimising the prescribing of drugs that may cause dependency: an evidence and gap map of systematic reviews**
Shaw L, et al

| Focus/Aim | Study (First author, date) | Type of Review (design of studies included), synthesis methods | Eligible age of participants within studies included in the review | Medications of interest | Medical condition for which medication of interest being taken | Relevance of systematic review to aim of evidence and gap map* | CEESAT set | Other comments |
|---|---|---|---|---|---|---|---|---|
| Evaluating intervention: optimise prescribing<br>Evaluating intervention: enhance adherence | King 2018 | SR (Quantitative: RCT, observational), Narrative | NR | Antidepressants | Depression | Medium | 2 | |
| Evaluating intervention: optimise prescribing | Kunstler 2019 | Rapid review (Quantitative: Systematic and narrative reviews), Narrative | NR | Benzodiazepines<br>Opiates<br>Antidepressants | Insomnia<br>Depression<br>Anxiety<br>Other<br>*Associated with following medication: ephalexin, propoxyphene, and cerebral and peripheral vasodilators*<br>NR | Medium | 3 | |

Under review

*Journal of Health Services Research & Policy*
**Optimising the prescribing of drugs that may cause dependency: an evidence and gap map of systematic reviews**
Shaw L, et al

| **Focus/Aim** | **Study (First author, date)** | **Type of Review (design of studies included), synthesis methods** | **Eligible age of participants within studies included in the review** | **Medications of interest** | **Medical condition for which medication of interest being taken** | **Relevance of systematic review to aim of evidence and gap map*** | **CEESAT set** | **Other comments** |
|---|---|---|---|---|---|---|---|---|
| Evaluating intervention: optimise prescribing | Loganathan 2011 | SR (Quantitative: RCTs, non-RCTs), Narrative | 65 years or over | Benzodiazepines | NR | Medium | 3 | Only a couple of included studies with medications of interest |
| Evaluating intervention: optimise prescribing | Low 2015 | SR (Quantitative: RCTs and quasi-experimental CTs), Narrative | Other *Older people* | Benzodiazepines Hypnotics/Z-drugs Antidepressants | NR | Low | 1 | |
| Evaluating intervention: deprescribing | Lynch 2020 | SR (Quantitative: RCTS), Narrative+ MA | 18 years or over | Benzodiazepines Hypnotics/Z-drugs | Insomnia Anxiety | High | 1 | |
| Evaluating intervention: deprescribing Evaluating intervention: optimise prescribing | Mathieson 2020 | SR (Quantitative: RCTs), Narrative | 18 years or over | Opiates | Chronic pain | High | 1 | |

Under Review

*Journal of Health Services Research & Policy*
**Optimising the prescribing of drugs that may cause dependency: an evidence and gap map of systematic reviews**
Shaw L, et al

| Focus/Aim | Study (First author, date) | Type of Review (design of studies included), synthesis methods | Eligible age of participants within studies included in the review | Medications of interest | Medical condition for which medication of interest being taken | Relevance of systematic review to aim of evidence and gap map* | CEESAT set | Other comments |
|---|---|---|---|---|---|---|---|---|
| Qualitative: other | Maund 2019a | SR (Qualitative: interviews, focus groups), Thematic | 18 years or over | Antidepressants | Depression | High | 1 | Insufficient data from a health professional perspective |

Under review

*Journal of Health Services Research & Policy*
**Optimising the prescribing of drugs that may cause dependency: an evidence and gap map of systematic reviews**
Shaw L, et al

| **Focus/Aim** | **Study (First author, date)** | **Type of Review (design of studies included), synthesis methods** | **Eligible age of participants within studies included in the review** | **Medications of interest** | **Medical condition for which medication of interest being taken** | **Relevance of systematic review to aim of evidence and gap map*** | **CEESAT set** | **Other comments** |
|---|---|---|---|---|---|---|---|---|
| Evaluating intervention: deprescribing | Maund, 2019b | SR (Quantitative: RCTs, cluster RCTs, quasi-experimental (non-RCT, BA, observational), Narrative+MA | 18 years or over | Antidepressants | Depression Anxiety | High | 1 | |
| Evaluating intervention: deprescribing Evaluating intervention: optimise prescribing | Mauri 2020 | Scoping review (Quantitative: ITS with/without comparison, controlled pre-post, uncontrolled pre-post, uncontrolled post-only, cross-sectional), Narrative | NR | Opiates | NR | Low | 3 | Includes some studies evaluating effectiveness of substitution treatment. Includes populations addicted to heroin |
| Guidelines Evaluating intervention: optimise prescribing Secondary aim | Mayer 2010 | SR (Guidelines), Narrative | Other *Physically active adults* | Opiates | Chronic pain | Medium | 3 | Focus on optimising prescribing secondary aim, drugs not of interest included |

Under review

*Journal of Health Services Research & Policy*
**Optimising the prescribing of drugs that may cause dependency: an evidence and gap map of systematic reviews**
Shaw L, et al

| Focus/Aim | Study (First author, date) | Type of Review (design of studies included), synthesis methods | Eligible age of participants within studies included in the review | Medications of interest | Medical condition for which medication of interest being taken | Relevance of systematic review to aim of evidence and gap map* | CEESAT set | Other comments |
|---|---|---|---|---|---|---|---|---|
| Evaluating intervention: enhance adherence | Milosavljevic 2018 | SR (Quantitative: RCTs, quasi-controlled trials, cluster-controlled trials, BA, retrospective/prospective cohort, observational, single group pre/post comparisons, statistical predictions), Narrative | NR | Antidepressants | Depression | Medium | 3 | |
| Evaluating intervention: optimise prescribing 'initiating treatment' | Moise 2018 | SR (Quantitative: RCTs, pre-post design studies, Narrative | 18 years or over | Antidepressants | Depression | Medium | 1 | Focus is about initiating treatment, of which only a small part is treatment with antidepressants |
| Evaluating intervention: optimise prescribing Evaluating intervention: deprescribing | Mokhar 2018 | SR (Quantitative: controlled design), Narrative | Other *Middle-aged adults (45 years and older)* | Benzodiazepines Hypnotics/Z-drugs | NR | High | 2 | |

Under review

*Journal of Health Services Research & Policy*
**Optimising the prescribing of drugs that may cause dependency: an evidence and gap map of systematic reviews**
Shaw L, et al

| Focus/Aim | Study (First author, date) | Type of Review (design of studies included), synthesis methods | Eligible age of participants within studies included in the review | Medications of interest | Medical condition for which medication of interest being taken | Relevance of systematic review to aim of evidence and gap map* | CEESAT set | Other comments |
|---|---|---|---|---|---|---|---|---|
| Evaluating intervention: deprescribing | Morera-Fumero 2020 | SR (Quantitative: cohort prospective, placebo-control double blind, double blind placebo-control cross-over), Narrative | NR | Benzodiazepines Hypnotics/Z-drugs | Insomnia | High | 2 | Intervention focusing on facilitating withdrawal |
| Evaluating intervention: deprescribing | Mugunthan 2011 | SR (Quantitative: RCTs), MA | 18 years or over | Benzodiazepines | NR | High | 2 | |
| Evaluating intervention: optimise prescribing Guidelines | Nguyen 2020 | SR (CPGs), Narrative | NR | Benzodiazepines Antidepressants | Anxiety Depression PTSD BPD Addiction | Medium | 3 | Included guidelines considered medication use in children |
| Evaluating intervention: enhance adherence | Nieuwlaat 2014 | SR (Quantitative: RCTs), Narrative | NR *Included studies characteristics table indicates both adult and child populations included* | Antidepressants | Depression Anxiety | Medium | 1 | |

Under review

*Journal of Health Services Research & Policy*
**Optimising the prescribing of drugs that may cause dependency: an evidence and gap map of systematic reviews**
Shaw L, et al

| **Focus/Aim** | **Study (First author, date)** | **Type of Review (design of studies included), synthesis methods** | **Eligible age of participants within studies included in the review** | **Medications of interest** | **Medical condition for which medication of interest being taken** | **Relevance of systematic review to aim of evidence and gap map*** | **CEESAT set** | **Other comments** |
|---|---|---|---|---|---|---|---|---|
| Guidelines Evaluating intervention: optimise prescribing | Nuckols 2014 | SR (Guidelines), Narrative | Adults | Opiates | Chronic pain | High | 2 | Includes patients taking methadone (n=12) |
| Evaluating intervention: enhance adherence Secondary aim | Nussbaumer 2014 | SR (Quantitative: RCTs, placebo-controlled, observational), Network MA | Adult 19+ years | Antidepressants | Depression | High | 2 | |
| Evaluating intervention: deprescribing | Ostini 2011 | SR (Quantitative: experimental and quasi-experimental research. RCTs, non-RCTs, CBA), Narrative | NR | Benzodiazepines Opiates | NR | Medium | 2 | |
| Evaluating intervention: deprescribing | Page 2016 | SR (Quantitative: RCTs, quasi-randomized controlled studies, non-RCTs, prospective and retrospective cohort, case-control, single arm, BA), MA | 65 years or over | Benzodiazepines Antidepressants | Insomnia Depression Anxiety | Medium | 2 | |

Under review

*Journal of Health Services Research & Policy*

**Optimising the prescribing of drugs that may cause dependency: an evidence and gap map of systematic reviews**

Shaw L, et al

| Focus/Aim | Study (First author, date) | Type of Review (design of studies included), synthesis methods | Eligible age of participants within studies included in the review | Medications of interest | Medical condition for which medication of interest being taken | Relevance of systematic review to aim of evidence and gap map* | CEESAT set | Other comments |
|---|---|---|---|---|---|---|---|---|
| Evaluating intervention: optimise prescribing<br>One of a variety of aims | Pantoja 2019 | SR (Quantitative: RCTs, non-RCTs), Narrative | NR | Benzodiazepines<br>Hypnotics/Z-drugs | NR<br>*Unclear why benzodiazepine prescribed* | Medium | 1 | Some included studies included child participants. Low amount of information about prescribing drugs of interest. Intervention is more of a general aid |
| Evaluating intervention: enhance adherence | Park 2017 | SR (Quantitative: Original research, comparison group, pre/post comparisons, statistical predictions), Narrative | NR | Antidepressants | Depression | Low | 3 | Not relevant to UK, medications not of interest included |
| Evaluating intervention: enhance adherence | Polinski 2011 | SR (Quantitative: self-report surveys or prescription drug claims"<br>Narrative | NR | Benzodiazepines<br>Antidepressants<br>Hypnotics/Z-drugs (sedatives) | Depression<br>Anxiety<br>NR<br>*Not clearly reported* | Low | 3 | Low relevance to UK. Other medications not of interest included. Note that focus is on 'use' which includes initiating prescribing. Drugs of interest may be currently under- or over-used |

Under review

*Journal of Health Services Research & Policy*
**Optimising the prescribing of drugs that may cause dependency: an evidence and gap map of systematic reviews**
Shaw L, et al

| **Focus/Aim** | **Study (First author, date)** | **Type of Review (design of studies included), synthesis methods** | **Eligible age of participants within studies included in the review** | **Medications of interest** | **Medical condition for which medication of interest being taken** | **Relevance of systematic review to aim of evidence and gap map*** | **CEESAT set** | **Other comments** |
|---|---|---|---|---|---|---|---|---|
| Evaluating intervention: deprescribing | Pollmann 2015 | Scoping review (Quantitative: Original investigations, research syntheses, guidelines, and narrative review articles), Narrative | 18 years or over | Benzodiazepines Hypnotics/Z-drugs | Insomnia Anxiety Other *Panic and "Mixed"* | High | 3 | |
| Evaluating intervention: enhance adherence Secondary aim: interventions to affect behaviour change that leads to prevention of chronic disease. | Posadzki 2016 | SR (Quantitative: RCTs, cluster RCTs, quasi-RCTs, ITS, CBA), Narrative+MA | NR | Opiates Antidepressants | Chronic pain Depression Anxiety | Medium | 1 | Included children. Interventions target general positive behaviour change in terms of health management. However there are specific studies reporting adherence-type interventions. Several non-relevant drugs and outcomes within the review |
| Qualitative: other: explored medication-taking experiences in patients, citizens, carers, relatives and clinicians | Rashid 2018 | SR (Qualitative: All types of qualitative studies, including those linked to observational, experimental, mixed methods papers. Interviews, focus groups or a combination), Thematic | Other | Antidepressants | Depression | Medium | 3 | Includes children and adults. Other medications not of interest included |

Under review

*Journal of Health Services Research & Policy*
**Optimising the prescribing of drugs that may cause dependency: an evidence and gap map of systematic reviews**
Shaw L, et al

| **Focus/Aim** | **Study (First author, date)** | **Type of Review (design of studies included), synthesis methods** | **Eligible age of participants within studies included in the review** | **Medications of interest** | **Medical condition for which medication of interest being taken** | **Relevance of systematic review to aim of evidence and gap map*** | **CEESAT set** | **Other comments** |
|---|---|---|---|---|---|---|---|---|
| Evaluating intervention: enhance adherence | Readdean 2018 | SR (Quantitative: controlled trials (both randomized and non-randomized), MA | 17 years or over | Antidepressants | Depression | High | 2 | |
| Patient views: intervention to encourage deprescribing Family/carer views: intervention to encourage deprescribing | Reeve 2013 | SR (Mixed: No limits placed on the type of methods used, qualitative, quantitative or mixed), Narrative/Content analysis/Thematic | Other *No limits were placed on the age of the participants* | Benzodiazepines Antidepressants | Depression Anxiety NR | Medium | 3 | Methods of synthesis unclear |
| Evaluating intervention: deprescribing | Reeve 2017 | SR (Quantitative: RCTs, non-RCTs), Narrative | 65 years or over | Benzodiazepines Hypnotics/Z-drugs | NR | High | 2 | |
| Evaluating intervention: enhance adherence | Rubio-Valera 2011 | SR (Quantitative: RCTs), MA | NR *Where reported, mean age within included studies ranged from 38-54 years* | Antidepressants | Depression | High | 2 | |

Under Review

*Journal of Health Services Research & Policy*

**Optimising the prescribing of drugs that may cause dependency: an evidence and gap map of systematic reviews**

Shaw L, et al

| Focus/Aim | Study (First author, date) | Type of Review (design of studies included), synthesis methods | Eligible age of participants within studies included in the review | Medications of interest | Medical condition for which medication of interest being taken | Relevance of systematic review to aim of evidence and gap map* | CEESAT set | Other comments |
|---|---|---|---|---|---|---|---|---|
| Practitioner views: prescribing medication of interest | Sirdifield 2013 | SR (Qualitative: focus groups, semi-structured interviews, GPs completed AUDIT and estimated own prescribing volume, observations, questionnaire for respondent characteristics), Thematic | NR | Benzodiazepines | Anxiety | High | 2 | |
| Qualitative: other Experiences and Perceptions of Seeking and Using Benzodiazepines and Z-Drugs | Sirdifield 2017 | SR (Qualitative: included if they involved qualitative analysis of patient experiences. Semi-structured interviews, focus groups), Thematic | NR<br>*In included studies, where reported, patient age ranged from 18-91, although focus primarily on older adults* | Benzodiazepines<br>Hypnotics/Z-drugs | Insomnia<br>Anxiety | High | 3 | |
| Evaluating intervention: optimise prescribing | Tedesco 2017 | SR (Quantitative: RCTs), MA | 18 years or over | Opiates | Other<br>*Pain management after TKA* | High | 1 | |

*Journal of Health Services Research & Policy*
**Optimising the prescribing of drugs that may cause dependency: an evidence and gap map of systematic reviews**
Shaw L, et al

| Focus/Aim | Study (First author, date) | Type of Review (design of studies included), synthesis methods | Eligible age of participants within studies included in the review | Medications of interest | Medical condition for which medication of interest being taken | Relevance of systematic review to aim of evidence and gap map* | CEESAT set | Other comments |
|---|---|---|---|---|---|---|---|---|
| Practitioner views: prescribing medication of interest | Toye 2017 | SR (Qualitative: Focus groups, semi-structured interview, open ended telephone interviews), Meta-ethnography | Other<br>*Adults* | Opiates | Chronic pain | High | 3 | |
| Evaluating intervention: optimise prescribing | Weatherburn 2020 | SR (Quantitative: ITS), MA<br>and regression | NR | Antidepressants | Depression | Medium | 3 | Some studies included children |
| Evaluating intervention: optimise prescribing | Wetzel 2018 | SR (Quantitative: pre/post intervention comparison, controlled clinical studies, time-series, compared post-intervention results with a predetermined baseline), Narrative | NR<br>*Where reported mean age within included studies ranged from 4.1 to 61.5 years* | Opiates | Other<br>*Post-operative pain* | High | 3 | One study focused on paediatric population. |
| Evaluating intervention: optimise prescribing | Wilbur 2018 | SR (Quantitative: RCTs and observational studies (judging by planned QA tools)Narrative | NR | Opiates | NR | Medium | 2 | Only two studies about relevant drug |

Under Review

*Journal of Health Services Research & Policy*
**Optimising the prescribing of drugs that may cause dependency: an evidence and gap map of systematic reviews**
Shaw L, et al

| Focus/Aim | Study (First author, date) | Type of Review (design of studies included), synthesis methods | Eligible age of participants within studies included in the review | Medications of interest | Medical condition for which medication of interest being taken | Relevance of systematic review to aim of evidence and gap map* | CEESAT set | Other comments |
|---|---|---|---|---|---|---|---|---|
| Evaluating intervention: optimise prescribing | Wilson 2019 | SR (Quantitative: pre-post, controlled before/after, case control, ITS, or cluster RCT), Narrative | NR | Opiates | Chronic pain | High | 2 | Includes individuals prescribed methadone |
| Evaluating intervention: optimise prescribing Secondary aim | Wong 2020 | SR (Quantitative: Experimental and observational studies. RCTs, non-controlled before-after studies), Narrative | 18 years or over | Opiates | Chronic pain | Medium | 2 | |
| Guidelines | Zhang 2020b | SR (CPGs, original studies, websites, standards, guides, protocols, statements), Narrative | Recommendations relevant to opioid-naive adults undergoing abdominopelvic surgery | Opiates | Other *Discharge after abdominopelvic surgery* | High | 1 | |
| Evaluating intervention: optimise prescribing | Zhang 2020a | SR (Quantitative: RCTs, nonrandomized trials, pre-post studies, ITS, cohort studies, case-control, historically controlled, cross-sectional), Narrative | 18 years or over | Opiates | Other *Individuals undergoing any type of surgery* | High | 1 | |

Under review

*Journal of Health Services Research & Policy*
**Optimising the prescribing of drugs that may cause dependency: an evidence and gap map of systematic reviews**
Shaw L, et al

Green highlighted text=High overall quality as appraised by AMSTAR-2, Turquoise highlighted text=Medium overall quality as appraised by AMSTAR-2, Orange highlighted text=Low overall quality as appraised by AMSTAR-2, Red highlighted text=Critically-low quality as appraised by the AMSTAR-2. *CEESAT Set: 1=prioritised for full quality appraisal using AMSTAR-2, CEESAT Set 2=Scored poorly on 1 out of 4 items on CEESAT, CEESAT Set 3=Scored poorly on 2-4 items on CEESAT. BA=Before and After, BPD=Borderline Personality Disorder, CBA=Controlled Before and After, CPG=Clinical Practice Guidelines, CT=Controlled-Trials, FH=Familial Hypercholesterolemia, ITS=Interrupted Time Series, MA=Meta-analysis, MMT=Methadone Maintenance Treatment, NR=Not Reported, OUD=Opioid Use Disorder, PPI=Proton Pump Inhibitors, PTSD=Post-Traumatic Stress Disorder, RCT=Randomized Controlled Trial, RQ=Research Question, SR=Systematic Review

Under Review

*Journal of Health Services Research & Policy*
**Optimising the prescribing of drugs that may cause dependency: an evidence and gap map of systematic reviews**
Shaw L, et al

*Table 4: Systematic reviews appraised using AMSTAR-2 which synthesised studies evaluating the effectiveness of an intervention*

Key for interpreting tables

Green highlighted text: High overall quality as appraised by AMSTAR-2

Turquoise highlighted text: Medium overall quality as appraised by AMSTAR-2

Orange highlighted text: Low overall quality as appraised by AMSTAR-2

Red highlighted text: Critically-low quality as appraised by the AMSTAR-2

CEESAT Set 1: Prioritised for full quality appraisal using AMSTAR-2

CEESAT Set 2: Scored poorly on 1 out of 4 items on CEESAT

CEESAT Set 3: Scored poorly on 2-4 items on CEESAT

| Focus/ Aim | Study (First author, date) | Medications of interest | Other medications included | Intervention name/s and aim | Outcomes measured | Setting/Context | Relevant part of care pathway |
|---|---|---|---|---|---|---|---|
| Evaluating intervention: deprescribing | Baandrup 2018 | Benzodiazepines Hypnotics/Z-drugs | NA | Pharmacological interventions to facilitate benzodiazepine withdrawal or to switch from benzodiazepine treatment to another drug | Benzodiazepine cessation, withdrawal symptoms, SAEs, Benzodiazepine mean dose, insomnia, anxiety, comorbid substance abuse, non-SAEs, relapse to benzodiazepine use, discontinuation due to AE | Primary/ health/social care Secondary health/social care (not hospital) Hospital (inpatient) Hospital (outpatient) | Discontinuing treatment |
| Evaluati ng interven tion: | Gould 2014 | Benzodiazepines | NA | Interventions for changing/reducing benzodiazepine prescribing: supervised gradual withdrawal, supervised abrupt withdrawal, both gradual and abrupt withdrawal, gradual withdrawal with a prescribing intervention, Withdrawal augmented with pharmacotherapy or psychotherapy. Prescription | Odds ratio in relation to not using benzodiazepines, at the level of patients or prescriptions | Primary/ health/social care Hospital (inpatient) Hospital (outpatient) Care home Patient home | Discontinuing treatment |

*Journal of Health Services Research & Policy*
**Optimising the prescribing of drugs that may cause dependency: an evidence and gap map of systematic reviews**
Shaw L, et al

| Focus/ Aim | Study (First author, date) | Medications of interest | Other medications included | Intervention name/s and aim | Outcomes measured | Setting/Context | Relevant part of care pathway |
|---|---|---|---|---|---|---|---|
| | | | | interventions: education, medication reviews, provision of prescribing feedback | | | |
| Evaluating intervention: deprescribing | Dills 2018 | Benzodiazepines Z-drugs Antidepressants | Diuretics, salmeterol, insulin, sulfonylurea, antipsychotics, anxiolytic, CV drugs, levodopa, nitrate, statins, antihypertensives | Deprescription: reduce medication burden, and enhance control of chronic medical and mental health conditions commonly managed by primary care physicians, compared with standard care in the non-terminally ill adult population | Primary outcome: successful deprescription, Secondary outcome: AE related to drug or underlying chronic condition as a result of deprescription | Primary/health /social care Hospital (inpatient) Hospital (outpatient) Care home Patient home | Discontinuing treatment |
| Evaluating intervention: deprescribing | Eccleston 2017 | Opiates | NA | Pharmacological, physiological, psychological or spiritual interventions to reduce/cease prescribed opioid use for the management of chronic non-cancer pain in adults: e.g. opioid antagonist treatment, dose tapering, or opioid replacement, physical therapy, massage, disability management, complementary therapies, or psychological approaches (CBT, counselling, and coping techniques) | Primary outcomes: prescribed opioid use in adults, AE related to opioid reduction, Secondary outcomes: pain intensity/severity, psychological functioning, physical functioning | NR | Discontinuing treatment |

*Journal of Health Services Research & Policy*
**Optimising the prescribing of drugs that may cause dependency: an evidence and gap map of systematic reviews**
Shaw L, et al

| Focus/ Aim | Study (First author, date) | Medications of interest | Other medications included | Intervention name/s and aim | Outcomes measured | Setting/Context | Relevant part of care pathway |
|---|---|---|---|---|---|---|---|
| Evaluating intervention: deprescribing | Hoyle 2018 | Benzodiazepines | Antipsychotics | Interventions to reduce antipsychotic and/or benzodiazepine use in nursing homes. Interventions included: education (meetings, distribution of educational materials, educational outreach), referral of patients to psychiatric support service, development of specific care plans, multicomponent interventions (all incorporated educational outreach for care staff) | Change in antipsychotic and/or benzodiazepine use, impact on clinical and/or economic outcomes | Care home | Discontinuing treatment |
| Evaluating intervention: deprescribing | Lynch 2020 | Benzodiazepines Hypnotics/Z-drugs | NA | Interventions to change patient's long-term BZRA use. All interventions advocated GDR to patients. Included: written letters signed by patients, prescribers or a clinical pharmacist, short consultations provided by health-care professionals [general practitioners (GPs), practice pharmacists, practice nurses] recommending reduction/discontinuation of the medications, empowerment-based personalized educational booklet posted to patients | Primary outcome: BZRA use (complete discontinuation or reduction by 25%) Secondary outcomes: health-related QOL; withdrawal symptoms; anxiety; sleep quality; depression; and health-care utilization (i.e. GP visits, hospital admissions, use of other medications) | Primary/health/ social care | Discontinuing treatment |

*Journal of Health Services Research & Policy*
**Optimising the prescribing of drugs that may cause dependency: an evidence and gap map of systematic reviews**
Shaw L, et al

| Focus/ Aim | Study (First author, date) | Medications of interest | Other medications included | Intervention name/s and aim | Outcomes measured | Setting/Context | Relevant part of care pathway |
|---|---|---|---|---|---|---|---|
| Evaluating intervention: deprescribing | Maund 2019b | Antidepressants | NA | Interventions to facilitate discontinuation of antidepressants: guided review of patients by primary care clinicians, abrupt discontinuation, tapering, psychological therapies, and pharmacologic approaches<br><br>e.g. mindfulness-based cognitive therapy, mindfulness-based cognitive therapy+tapering support, CBT, Guided primary care clinician review, patient-specific letters to primary care clinician recommending to discontinue antidepressant and provide tapering advice, CBT+ tapering, MBCT+tapering, gradual discontinuation, 1 week of tapering | Discontinuation of antidepressants, discontinuation symptoms, relapse/recurrence, QOL, antidepressant reduction, sexual function; other outcomes (e.g., social and occupational function, well-being, quality of relationships) | Primary/health/ social care<br>Secondary health/social care (not hospital) | Discontinuing treatment |
| Evaluating intervention: deprescribing | Al Shemeili 2015 | Hypnotics/Z-drugs | Anticholinergic | Drug Burden Index to identify potentially inappropriate prescribing of anticholinergic and sedative agents in elderly patients in institutionalised care | Prescribing of anticholinergic and sedative agents (e.g. cessation of therapy, prescribing altered to other agents, reduction in adverse drug reactions), DBI scores, physical and mental functioning; adverse effects of anticholinergic and sedative medicines, changes to therapy following application of DBI | Hospital (inpatient)<br>Care home | Discontinuing treatment |
| Evaluati ng interven tion: | Brown 2019 | Antidepressants | NA | Pharmacy-based management interventions to improve depression outcomes | Depression symptom level, Acceptability of the intervention, diagnosis of depression, non- | Primary/health/ social care<br>Secondary health/social care | Pre-treatment/ Initiation<br>Maintaining treatment |

*Journal of Health Services Research & Policy*
**Optimising the prescribing of drugs that may cause dependency: an evidence and gap map of systematic reviews**
Shaw L, et al

| Focus/ Aim | Study (First author, date) | Medications of interest | Other medications included | Intervention name/s and aim | Outcomes measured | Setting/Context | Relevant part of care pathway |
|---|---|---|---|---|---|---|---|
| enhance adherence | | | | | adherence to medication, frequency of primary care appointments, QOL, social functioning, AE | (not hospital) Hospital (inpatient) Hospital (outpatient) | |
| Evaluating intervention: enhance adherence | Garcia-Perez 2011 | Antidepressants | Antipsychotics, bipolar medication | Interventions to increase medication adherence, including: psychosis compliance therapy, collaborative care, compliance enhancing intervention, pharmacy-based intervention, financial incentive | Medication adherence | Primary/health/ social care Secondary health/social care (not hospital) Hospital (inpatient) | Maintaining treatment |
| Evaluating intervention: | Posadzki 2016 | Opiates Antidepressants | Asthma medication, prescription drugs taken without prescription or more than prescribed, illicit non-injection drugs, glaucoma medications, inhaled corticosteroids, insulin, methadone maintenance, cholinesterase inhibitor medications, oral hypoglycaemic medications, nicotine replacement therapy, bupropion, and varenicline, antiretroviral treatment, bone active medication, non-hormonal oral agents, statins, antihypertensives | Automated telephone communication systems for preventing disease and managing long-term conditions | Health behaviour and clinical outcomes, changes in health-enhancing behaviour, risk-taking behaviour, physiological measures, blood biochemistry. Process outcomes: change in acceptability of service (e.g. consumer accessibility/usability of interventions to apply information/support supplied), satisfaction (e.g. patient/carer) the intervention), cost-effectiveness. Cognitive outcomes: changes in knowledge (i.e. accurate risk knowledge and perception), attitude and intention to change, self-efficacy. Patient-centred outcomes: quality of life, adverse outcomes, | Primary/health/ social care Secondary health/social care (not hospital) Hospital (inpatient) Hospital (outpatient) Patient home | Pre-treatment/ Initiation Maintaining treatment |

*Journal of Health Services Research & Policy*
**Optimising the prescribing of drugs that may cause dependency: an evidence and gap map of systematic reviews**
Shaw L, et al

| Focus/ Aim | Study (First author, date) | Medications of interest | Other medications included | Intervention name/s and aim | Outcomes measured | Setting/Context | Relevant part of care pathway |
|---|---|---|---|---|---|---|---|
| | | | | | unintended AE attributable to intervention | | |
| Evaluating intervention: | Nieuwlaat 2014 | Antidepressants | Medications to treat following conditions: HIV/AIDS, psychiatric disorders, chronic obstructive pulmonary disease, CVD/CV risk, diabetes, antibiotics, arthritis, complex chronic care, dyspepsia, glaucoma, oral anticoagulation, osteoporosis, tuberculosis, acne, cancer, hepatitis, iron supplementation during pregnancy, liver transplant, malaria, oral contraceptives, | Interventions to affect adherence with prescribed, self-administered medications | Medication adherence, clinical outcomes | Primary/health/ social care<br>Hospital (inpatient)<br>Hospital (outpatient)<br>Care home<br>Patient home | Maintaining treatment |

*Journal of Health Services Research & Policy*

**Optimising the prescribing of drugs that may cause dependency: an evidence and gap map of systematic reviews**

Shaw L, et al

| Focus/ Aim | Study (First author, date) | Medications of interest | Other medications included | Intervention name/s and aim | Outcomes measured | Setting/Context | Relevant part of care pathway |
|---|---|---|---|---|---|---|---|
| | | | tonsillectomy/adenoidectomy, ulcerative colitis, statins, antihypertensives | | | | |
| Evaluating intervention: optimise prescribing | Gillaizeau 2013 | Antidepressants | Insulin, aminoglycoside antibiotics, anticoagulants, theophylline, anti-rejection drugs, anaesthetic agents, gonadotropins | Computerized advice on drug dosage to improve prescribing practice computerized advice on drug dosage: optimize effects and minimize dangers | Proportion of participants/time: plasma drug concentrations within therapeutic range, studied physiological parameter within the target range. "Time to achieve therapeutic control, proportion of participants: with toxic drug levels, with clinical improvement, with adverse effects of drug therapy. Proportion of deaths, LOS, total cost/participant | Primary/health /social care Secondary health/social care (not hospital) Hospital (inpatient) Hospital (outpatient) | Pre-treatment/ Initiation Maintaining treatment |

Under

*Journal of Health Services Research & Policy*
**Optimising the prescribing of drugs that may cause dependency: an evidence and gap map of systematic reviews**
Shaw L, et al

| Focus/ Aim | Study (First author, date) | Medications of interest | Other medications included | Intervention name/s and aim | Outcomes measured | Setting/Context | Relevant part of care pathway |
|---|---|---|---|---|---|---|---|
| Evaluating intervention: optimise prescribing | Hukins 2019 | Benzodiazepines Hypnotics/Z-drugs | Anticholinergic medications, anxiolytics, oestrogens, NSAID, antipsychotics, antiplatelets, proton pump inhibitors, statins | Any tool (dementia-specific or non-disease-specific) to identify potentially inappropriate prescribing | Prevalence of polypharmacy, prevalence of PIP, most common prescribed PIP medications | Primary/health/ social care Secondary health/social care (not hospital) Hospital (inpatient) Hospital (outpatient) Care home Patient home | Discontinuing treatment |
| Evaluating intervention: optimise prescribing | Low 2015 | Benzodiazepines Hypnotics/Z-drugs Antidepressants | Antipsychotics, NSAID | Interventions to change the care practices of staff for the benefit of the residents. Categorized interventions via their components: educational material (written material or a DVD/video or online website), training: delivered in person to staff, reminders (e.g. postcards, posters) designed to prompt practice, audit and feedback, mentoring/support (supervision/consultation/mentoring of staff in teams or individually), champions responsible for driving change, consensus/multidisciplinary team meetings to discuss issues relating to practice, policy/procedure, change to staff responsibilities of staff or care organisation | Change in staff behaviour, change in other staff outcomes (e.g. staff turnover, absenteeism, stress), change in resident clinical outcomes (not just satisfaction with care). Hygiene and infection control, nutrition, nursing home acquired pneumonia, depression, appropriate prescribing, physical restraint reduction, management of behavioural/psychological symptoms of dementia, falls reduction/prevention, quality improvement, philosophy of care, advance care directive, pain management, assault reduction, resident to resident mistreatment, pressure ulcer reduction, oral health | Care home | Pre-treatment/ Initiation Discontinuing treatment |

*Journal of Health Services Research & Policy*
**Optimising the prescribing of drugs that may cause dependency: an evidence and gap map of systematic reviews**
Shaw L, et al

| Focus/ Aim | Study (First author, date) | Medications of interest | Other medications included | Intervention name/s and aim | Outcomes measured | Setting/Context | Relevant part of care pathway |
|---|---|---|---|---|---|---|---|
| Evaluating intervention: optimise prescribing | Tedesco 2017 | Opiates | NSAID | Non-pharmacological interventions to improve pain management after TKA: CPM, preoperative exercise, cryotherapy, electrotherapy, acupuncture | Postoperative pain relief, opioid/other analgesic consumption, time to first request for analgesia | Hospital (inpatient) | Pre-treatment/ Initiation Discontinuing treatment |
| Evaluating intervention: optimise prescribing | Zhang 2020b | Opiates | NA | Behavioural interventions and/or implementation strategies targeted at healthcare workers to change their behaviour, such as audit and feedback, educational meetings, local consensus processes, public release of performance data to reduce opioid prescription after surgery | Primary outcome: amount of opioid prescribed at hospital discharge after surgery. Secondary outcomes: postoperative pain control, overall satisfaction with pain management | Hospital (inpatient) | Pre-treatment/ Initiation |

*Journal of Health Services Research & Policy*
**Optimising the prescribing of drugs that may cause dependency: an evidence and gap map of systematic reviews**
Shaw L, et al

| Focus/ Aim | Study (First author, date) | Medications of interest | Other medications included | Intervention name/s and aim | Outcomes measured | Setting/Context | Relevant part of care pathway |
|---|---|---|---|---|---|---|---|
| Evaluating intervention: optimise prescribing | Moise 2018 | Antidepressants | NA<br>*Although several other non-pharmacological treatment modalities included* | Interventions to promote treatment initiation. Interventions included:<br>Cultural tailoring, motivation, treatment preference matching, case management, education, collaborative or integrated care, shared or clinical decision-making | Depression treatment initiation, treatment retention, mean change in depressive symptoms | Primary/health/ social care<br>Secondary health/social care (not hospital)<br>Hospital (outpatient)<br>Patient home (community) | Pre-treatment/ Initiation |
| Evaluating intervention: optimise prescribing* | Pantoja 2019 | Benzodiazepines<br>Hypnotics/Z-drugs | Anticonvulsants, antibiotic therapy, NSAID, nicotine gum, ezetimibe, thromboprophylaxis, statins, antihypertensives | Manual paper reminders to improve compliance with preventive guidelines and disease management guidelines | Patient-important endpoints: death, development of a pulmonary embolism , surrogate or intermediate endpoints: achievement of target blood pressure or serum cholesterol level, markers of disease/health status, adverse effects, resource use, changes in professional practice, patient outcomes | Primary/health /social care<br>Hospital (inpatient)<br>Hospital (outpatient) | Pre-treatment/ Initiation |
| Evaluating Intervention: | Mathieson 2020 | Opiates | NA | Interventions to reduce/cease prescription/ use of opioid analgesics in patients with chronic non-cancer pain: e.g. Dose reduction protocols, Opioid replacement with buprenorphine, mindfulness, Therapeutic Interactive Voice Response programme, meditation and CBT, electro acupuncture, Clinician-focused deprescribing | Daily dose, opioid analgesic prescriptions, proportion participants who ceased/reduced opioid use, SAE/AE, pain intensity, disability, quality-of-life | Primary/health/ social care<br>Secondary health/social care (not hospital)<br>Hospital (outpatient) | Discontinuing treatment |

*Journal of Health Services Research & Policy*

**Optimising the prescribing of drugs that may cause dependency: an evidence and gap map of systematic reviews**

Shaw L, et al

| Focus/ Aim | Study (First author, date) | Medications of interest | Other medications included | Intervention name/s and aim | Outcomes measured | Setting/Context | Relevant part of care pathway |
|---|---|---|---|---|---|---|---|
| prescrib ing | | | | interventions (education-based multicomponent intervention), decision tools to improve guideline adherence and decrease opioid misuse risk, online education of patient simulation+case-based learning for safer prescribing | | | |

*A secondary aim or one of multiple aims of review. Green highlighted text=High Overall Quality AMSTAR-2, Turquoise highlighted text=Medium Overall Quality AMSTAR-2, Orange highlighted text=Low Overall Quality AMSTAR-2, Red highlighted text=Critically-low Overall Quality AMSTAR-2. ACEI= Angiotensin-Converting Enzyme Inhibitors, AE=Adverse Events, AIDS= Acquired Immunodeficiency Syndrome, BEZRA=Benzodiazepines/Zdrugs, BP=Blood Pressure, CBT=Cognitive Behavioural Therapy, CPM=Continuous Passive Motion, CRT= Cardiac Resynchronisation Therapy, CV=Cardiovascular, CVD=Cardiovascular Disease, DBI=Drug Burden Index, ED=Emergency Department, GDR=Gradual Dose Reduction, GP=General Practitioner, HF=Heart Failure, HIV= Human Immunodeficiency Virus, HT=Hypertension, LDL= Low-Density Lipoprotein, LOS=Hospital Length of Stay, NA=Not Applicable, NR=Not Reported, QOL=Quality Of Life, LVEF= Left Ventricular Ejection Fraction, MBCBT=Mindfulness Based Cognitive Behavioural Therapy, NSAID=Non-Steroidal Anti-Inflammatories, PIP=Potentially Inappropriately Prescribed, SAE=Serious Adverse Events, SDM=Shared Decision Making, SMBP=Self-Monitoring of Blood Pressure, TKA=Total Knee Arthroplasty

der Review

*Journal of Health Services Research & Policy*
**Optimising the prescribing of drugs that may cause dependency: an evidence and gap map of systematic reviews**
Shaw L, et al

*Table 51: Systematic reviews scoring negatively on one CEESAT item which synthesised studies evaluating the effectiveness of an intervention*

| Focus/ Aim | Study (First author, date) | Medications of interest | Other medications included | Intervention name/s and aim | Outcomes measured | Setting/Context | Relevant part of care pathway |
|---|---|---|---|---|---|---|---|
| Evaluating intervention: deprescribing | Darker 2015 | Benzodiazepines | NA | Interventions to reduce BZ use: Psychosocial intervention, contingency management, community reinforcement approaches, CBTs, relapse prevention, couples based interventions, family- based interventions, psychodynamic therapies, drug abuse counselling, BIs, coping skills training, supportive expressive therapy, social skills training, stress management, relaxation therapy, relapse prevention, DBT, MI/motivational enhancement therapies | Successful discontinuation or reduction of BZ use by > 50%, metabolites, use of BZs, degree of effective dose reduction, abstinence rates, time to relapse, drop-outs/loss to follow-up | Primary/health/ social care Secondary health/social care (not hospital) Hospital (inpatient) Hospital (outpatient) | Discontinuing treatment |
| Evaluating intervention: deprescribing | Frank 2017 | Opiates | NA | Strategies to reduce or discontinue long term opioid therapy among adults prescribed LTOT for chronic pain: Interdisciplinary pain programs, buprenorphine-assisted dose reduction, behavioural interventions, other outpatient programs, other interventional programs, detoxification, ketamine-assisted dose reduction, acupuncture | Reduction/discontinuation LTOT. Patient outcomes: pain, pain-related function, quality of life, opioid withdrawal symptoms, new-onset substance use, adverse events, opioid- related overdose death | Primary/health /social care Hospital (inpatient) Hospital (outpatient) | Discontinuing treatment |
| Evaluating interventio n: [illegible] | Mugunthan 2011 | Benzodiazepines | NA | Minimal intervention (letter, self-help information, short consultation with a GP): reduce the long-term use of BZs in primary care | BZ use, general health status, adverse effects of withdrawal, other medicines used, psychiatric morbidity, insomnia | Primary/health/ social care | Discontinuing treatment |

*Journal of Health Services Research & Policy*
**Optimising the prescribing of drugs that may cause dependency: an evidence and gap map of systematic reviews**
Shaw L, et al

| Focus/ Aim | Study (First author, date) | Medications of interest | Other medications included | Intervention name/s and aim | Outcomes measured | Setting/Context | Relevant part of care pathway |
|---|---|---|---|---|---|---|---|
| Evaluating intervention: deprescribing | Ostini 2011 | Benzodiazepines Opiates | Antibiotics, metformin, proton pump inhibitors, hormone replacement therapy | Interventions to stop pre-existing prescribing in situations where continued prescribing may no longer be clinically warranted: patient mediated interventions, manual reminders to prescribers, educational materials given to patients, face-to-face intervention with individual prescribers, regulatory intervention, Audit and feedback, electronic reminders, educational materials sent to prescribers, educational meetings with prescribers+ distance communication | Stopping prescribing | Primary/health/ social care Patient home | Discontinuing treatment |

*Journal of Health Services Research & Policy*
**Optimising the prescribing of drugs that may cause dependency: an evidence and gap map of systematic reviews**
Shaw L, et al

| Focus/ Aim | Study (First author, date) | Medications of interest | Other medications included | Intervention name/s and aim | Outcomes measured | Setting/Context | Relevant part of care pathway |
|---|---|---|---|---|---|---|---|
| Evaluating intervention: deprescribing | Page 2016 | Benzodiazepines Antidepressants | Clopidogrel, Cilostazol, pentoxifylline, diuretics, nitrates, Glucosamine, bisphosphonates, calcium, vitamin D, Calcitriol, conjugated equine estrogens Premarin combined with medroxyprogesterone acetate, Carbamazepine, Levodopa, lithium, anticholinestera, prednisolone, corticosteroids, d beta-2 receptor agonist, antipsychotics, statins, antihypertensives | Deprescribing by a health care professional of one or more regular prescription medications: | Mortality, adverse drug withdrawal events, physical health, cognitive function and psychological health, quality of life | Hospital (inpatient) Care home Primary/health/ social care Hospital (outpatient) Patient home | Discontinuing treatment |

*Journal of Health Services Research & Policy*
**Optimising the prescribing of drugs that may cause dependency: an evidence and gap map of systematic reviews**
Shaw L, et al

| Focus/ Aim | Study (First author, date) | Medications of interest | Other medications included | Intervention name/s and aim | Outcomes measured | Setting/Context | Relevant part of care pathway |
|---|---|---|---|---|---|---|---|
| Evaluating intervention: deprescribing | Reeve 2017 | Benzodiazepines Hypnotics/Z-drugs | NA | Interventions to deprescribe benzodiazepines and other hypnotics among older people: GP-targeted intervention, pharmacological substitution, mixed interventions including temporary pharmacological substitution + psychological support, tapering with psychological support, tapering with patient education | Positive and negative (e.g. adverse drug withdrawal reactions) clinical outcomes, continuing vs. stopping BZ/Z-drug use | Primary/health/ social care Hospital (inpatient) Care home Patient home | Discontinuing treatment |
| Evaluating intervention: deprescribing | Morera-Fumero 2020 | Benzodiazepines Hypnotics/Z-drug | NA | Melatonin and melatonin agonists as treatments for benzodiazepines and hypnotics withdrawal in patients with primary insomnia | Detoxification at end of treatment | NR | Discontinuing treatment |
| Evaluating intervention: enhance | Al-Jumah 2012 | Antidepressants | NA | Pharmacist interventions: enhance patients adherence to antidepressant medication | Adherence, patient satisfaction, psychological dimensions (depressive symptoms, mood, knowledge) | Primary/health/ social care | Maintaining treatment |

*Journal of Health Services Research & Policy*
**Optimising the prescribing of drugs that may cause dependency: an evidence and gap map of systematic reviews**
Shaw L, et al

| Focus/ Aim | Study (First author, date) | Medications of interest | Other medications included | Intervention name/s and aim | Outcomes measured | Setting/Context | Relevant part of care pathway |
|---|---|---|---|---|---|---|---|
| Evaluating intervention: enhance adherence | Garcia-Perez 2020 | Antidepressants | Pychotropic prescription, anxiety or depression medication, antipsychotic | Interventions to improve medication adherence in mental health: Psychosis compliance therapy, multifaceted, stepped, collaborative care intervention, compliance-enhancing programme, pharmacy-based intervention, treatment supported by pharmacogenetics test, value-based benefit design policy: waived co-payments for medications, mental health counselling, promotion of a management programme for depression, financial incentive | Costs, adherence to medication therapy | Primary/health/social care<br>Secondary health/social care (not hospital)<br>Hospital (inpatient)<br>Hospital (outpatient) | Information and/or advice<br>Working with other health or social care services/ charities<br>Patient adherence<br>Progress review |
| Evaluating intervention: enhance adherence | Park 2017 | Antidepressants<br>Antihypertensives | Antidementia medication, pneumonia-related ambulatory antibiotics, antilpemics, insulin, biologics, oral diabetes drugs, antiasthmatics, pain reliever, phosphate binders, cinacalce, teriparatide, statins | Medicare Part D: increase drug utilization and lower Medicare and Medicaid participants out- of-pocket prescription drug costs, thereby improving access | Out-of-pocket costs, drug utilization | Primary/health/social care<br>Hospital (inpatient)<br>Hospital (outpatient) | Pre-treatment/ Initiation<br>Maintaining treatment |

*Journal of Health Services Research & Policy*
**Optimising the prescribing of drugs that may cause dependency: an evidence and gap map of systematic reviews**
Shaw L, et al

| Focus/ Aim | Study (First author, date) | Medications of interest | Other medications included | Intervention name/s and aim | Outcomes measured | Setting/Context | Relevant part of care pathway |
|---|---|---|---|---|---|---|---|
| Evaluating interventio n: enhance | Readdean 2018 | Antidepressants | NA | Pharmacist education and counselling interventions: improving antidepressant medication adherence and depression symptomology | Medication adherence, depression | Primary/health/soci al care<br>Hospital (outpatient) | Maintaining treatment |
| Evaluating intervention: enhance | Rubio-Valera 2011 | Antidepressants | NA | Pharmacist interventions to improve adherence to antidepressant medication among outpatients with depressive disorder: educational messages and counselling, monitoring and medication dosage adjustment and management of adverse effects | Adherence | Primary/health/soci al care<br>Secondary health/social care (not hospital)<br>Hospital (outpatient) | Maintaining treatment |
| Evaluating intervention: enhance adherence | Conn 2016 | Antidepressants | Yes, in relation to following medical conditions: gallbladder disease, unspecified chronic illnesses, osteoarthritis, asthma, IBD, glaucoma, kidney transplant, anti-coagulant therapy, osteoarthritis stroke, diabetes, HIV, statins, antihypertensives | Medication Adherence Interventions: Intervention content could include: prompts/cues to administer medications, self-monitoring of medication administration, self-monitoring of disease symptoms, written instructions, rewards for increased adherence, increased communication between providers and patients, providing feedback to participants about their adherence, goal setting about adherence, habit assessment/modification, and problem solving about adherence challenges | Adherence | Primary/health/soci al care<br>Secondary health/social care (not hospital)<br>Patient home | Maintaining treatment |

*Journal of Health Services Research & Policy*

**Optimising the prescribing of drugs that may cause dependency: an evidence and gap map of systematic reviews**

Shaw L, et al

| Focus/ Aim | Study (First author, date) | Medications of interest | Other medications included | Intervention name/s and aim | Outcomes measured | Setting/Context | Relevant part of care pathway |
|---|---|---|---|---|---|---|---|
| Evaluating intervention: enhance adherence | Milosavljevic 2018 | Antidepressants | Yes, in relation to following conditions: Asthma, diabetes, dyslipidemia, COPD, 'older people's health' aldosterone-inhibiting diuretics (for heart failure), intravenous vitamin D analogs, calcimimetic, oral hypoglycemics, antipsychotics, other central nervous system drugs, beta blockers, analgesics, antihypertensives | Community pharmacist interventions: improve adherence, improve clinical outcomes. | Behavioural outcome: adherence, self-efficacy. Clinical outcome: clinical biomarkers (e.g. blood pressure, glycosylated haemoglobin, LDL), hospitalisation rates, mortality, emergency room visits, markers of disease progress. Economic outcome: cost effectiveness analysis, or other relevant analysis. Humanistic outcome: patient quality of life, knowledge, or satisfaction | Primary/health/social care Patient home | Pre-treatment/ Initiation Maintaining treatment |
| Evaluating intervention: enhance | Nussbaumer 2014 | Antidepressants | NA | Extended-release antidepressants for medical management of MDD: improve adherence and a lower risk of adverse events | Comparative efficacy, risk of harms, adherence, response, remission, QOL, depression | NR | Pre-treatment/ Initiation Maintaining treatment |

*Journal of Health Services Research & Policy*
**Optimising the prescribing of drugs that may cause dependency: an evidence and gap map of systematic reviews**
Shaw L, et al

| Focus/ Aim | Study (First author, date) | Medications of interest | Other medications included | Intervention name/s and aim | Outcomes measured | Setting/Context | Relevant part of care pathway |
|---|---|---|---|---|---|---|---|
| Evaluating intervention: enhance adherence* | Huang 2013 | Antidepressants | Oral hypoglycaemic agents | Collaborative care including: multi-professional patient care, structured management plan, scheduled patient follow up, enhanced inter-professional communication<br><br>Aim: improve depression and diabetes outcomes in patients with both depression and diabetes | Depression treatment response, depression remission, haemoglobin A1c (HbA1c) control, adherence to medication (including adherence of oral hypoglycaemic agents and/or antidepressants) | Primary/health/social care | Maintaining treatment |
| Evaluating intervention: optimise prescribing | Awadalla 2020 | Opiates | NA | Stewardship Interventions, including: forcing functions and educational interventions (treatment algorithm forces the user into certain pathways to guide decision-making), prior authorization policies, order restrictions, education for both prescribers and patients. All interventions performed at a system level. Aim: optimise the prescribing of extended-release opioids for patients with acute pain | Prescribing of extended-release opioids | Primary/health/social care<br>Hospital (inpatient)<br>NR | Pre-treatment/ Initiation<br>Discontinuing treatment |
| Evaluating intervention: optimise [illegible] | Becker 2016 | Antidepressants | Psychiatric medication | Direct-to-Consumer Advertising: enhance patient awareness and education by providing legitimate information about conditions and treatment options | Patient requests for advertised medication, physician prescribing in response to patient requests | Primary/health/social care | Pre-treatment/ Initiation |

*Journal of Health Services Research & Policy*
**Optimising the prescribing of drugs that may cause dependency: an evidence and gap map of systematic reviews**
Shaw L, et al

| Focus/ Aim | Study (First author, date) | Medications of interest | Other medications included | Intervention name/s and aim | Outcomes measured | Setting/Context | Relevant part of care pathway |
|---|---|---|---|---|---|---|---|
| Evaluating intervention: optimise prescribing | Hopkins 2019 | Opiates | NA | Prescriber education interventions to optimize opioid prescribing in acute care: stand-alone education, face-to-face education within multifaceted interventions, incorporating other responsible prescribing strategies including new guidelines or consensus recommendations, continuous auditing and individualized feedback, and changes to computerized provider order entry (CPOE) systems | Medication use or prescribing | Hospital (inpatient) Hospital (outpatient) | Pre-treatment/ Initiation |
| Evaluating interventio n: optimise [illegible] | Wilbur 2018 | Opiates | Asthma inhalers | Standardised Patient training: Undergraduate medical training | Improve medical proficiency, of relevance optimised opioid prescribing, Knowledge, behaviours | Primary/health/soci al care | Pre-treatment/ Initiation |
| Evaluating intervention: optimise prescribing | Wilson 2019 | Opiates | Methadone | Prescription monitoring programs to reducing opioid prescribing, dispensing, and use: monitors outpatient prescription dispensing of opioids (or other drugs) by health care providers | Change in overall volume of opioids prescribed/dispensed or proportion of specific opioids (e.g., oxycodone, hydrocodone) prescribed/dispensed, change in rates of multiple provider use, change in rates of inappropriate prescribing or dispensing practices, change in rates of nonmedical prescription opioid use | NR | Pre-treatment/ Initiation Discontinuing treatment |

*Journal of Health Services Research & Policy*
**Optimising the prescribing of drugs that may cause dependency: an evidence and gap map of systematic reviews**
Shaw L, et al

| Focus/ Aim | Study (First author, date) | Medications of interest | Other medications included | Intervention name/s and aim | Outcomes measured | Setting/Context | Relevant part of care pathway |
|---|---|---|---|---|---|---|---|
| Evaluating intervention: optimise prescribing Evaluating intervention: [illegible] | Mokhar 2018 | Benzodiazepines Hypnotics/Z-drugs | NA | Patient-centred treatments to reduce BZ use and/or prescription: predominantly educational or informational" | BZ use and/or prescription | Primary/health/social care Secondary health/social care (not hospital) Hospital (inpatient) Hospital (outpatient) Care home | Discontinuing tratment |
| Evaluating intervention: optimise prescribing Guidelines | Beaudoin 2016 | Opiates | NA | Policies/guidelines aimed at Prescriber practices to guide opioid prescribing | Prescriber level: Opioid prescribing (as proportion of patient visits and of all prescriptions; total number of prescriptions; average quantity prescribed; total opioid prescriptions exceeding upper daily limit in guidelines). Patient level: negative health outcomes related to opioid use (e.g. deaths, overdoses, hospital visits) | Primary/health/social care Secondary health/social care (not hospital) Hospital (outpatient) | Pre-treatment/ Initition Prescribing guidelines |

*Journal of Health Services Research & Policy*
**Optimising the prescribing of drugs that may cause dependency: an evidence and gap map of systematic reviews**
Shaw L, et al

| Focus/ Aim | Study (First author, date) | Medications of interest | Other medications included | Intervention name/s and aim | Outcomes measured | Setting/Context | Relevant part of care pathway |
|---|---|---|---|---|---|---|---|
| Evaluating intervention: optimise prescribing* | Kimmel 2019 | Opiates | NA | Real-time electronic notifications, including predictive analytics and decision support applications: reduce ED recidivism | Healthcare utilization outcomes: inpatient and outpatient visits, 30-day hospital readmissions, ED or hospital length of stay, use of and prescription of opioids, use of diagnostic imaging and laboratory tests, healthcare cost, organizational financial performance, access to medical insurance, primary care, and safe housing access, ED recidivism risk | Hospital (inpatient) Hospital (outpatient) | Pre-treatment/ Initiation |
| Evaluating intervention: optimise prescribing* | Wong 2020 | Opiates | NA | ED-based interventions: four common intervention components: electronic medical record (EMR) alerts, primary care contact and referral, individualized care plans or care pathways, and departmental opioid restriction policies. Intervention aim: improve ED visit frequency as well as other patient-, physician-, and system-important outcomes | Frequency of ED visits. Secondary outcomes: amount and type of opioids administered in ED, amount and type of opioids pre- scribed at ED discharge, total care-associated costs (e.g., physician, pharmacy, hospital bills), AE | Hospital (outpatient) | Pre-treatment/ Initiation |

*Journal of Health Services Research & Policy*
**Optimising the prescribing of drugs that may cause dependency: an evidence and gap map of systematic reviews**
Shaw L, et al

| Focus/ Aim | Study (First author, date) | Medications of interest | Other medications included | Intervention name/s and aim | Outcomes measured | Setting/Context | Relevant part of care pathway |
|---|---|---|---|---|---|---|---|
| Evaluating intervention: optimise prescribing<br>Evaluating intervention: enhance | King 2018 | Antidepressants | Diabetic medication, Digoxin, mixed medications, statins, antihypertensives | Longer-duration (2–4 months) versus shorter-duration (28-day)prescriptions to improve patient outcomes | Health outcomes, adverse events, medication adherence, medication wastage, professional administration time, pharmacists time and/or costs, patient experience, and patient out-of-pocket costs | Primary/health/social care<br>Secondary health/social care (not hospital)<br>Hospital (inpatient) | Pre-treatment/ Initiation<br>Maintaining treatment |

*Secondary aim or outcome within the review. AE=Adverse Events, BI=Behavioural Interventions, BP=Blood Pressure, BZ=Benzodiazepine, CBT=Cognitive Behavioural Therapy, COPD=Coronary Obstructive Pulmonary Disease, DBT=Dialectical Behavioural Therapy, ED=Emergency Department, GP=General Practitioner, HIV= Human Immunodeficiency Virus, IBD=Irritable Bowel Disease, LDL= Low-Density Lipoprotein, LTOT=Long-Term Opioid Therapy, MDD=Major Depressive Disorder, MI=Motivational Interviewing, NA=Not Applicable, NR=Not Reported, QOL=Quality of Life, VBID=Value Based Insurance Design

Under Review

*Journal of Health Services Research & Policy*
**Optimising the prescribing of drugs that may cause dependency: an evidence and gap map of systematic reviews**
Shaw L, et al

*Table 62: Systematic reviews scoring negatively on 2-4 CEESAT items which synthesised studies evaluating the effectiveness of an intervention*

| Focus/ Aim | Study (First author, date) | Medications of interest | Other medications included | Intervention name/s and aim | Outcomes measured | Setting/Context | Relevant part of care pathway |
|---|---|---|---|---|---|---|---|
| Evaluating intervention: deprescribing | Pollmann 2015 | Benzodiazepines<br>Hypnotics/Z-drugs | NA | Interventions to discontinue benzodiazepines and sedative hypnotics in community-dwelling individuals: pharmacologic therapy, psychological therapy, mixed, other, gradual dose reduction | Direction of effect of endpoints related to benzodiazepine and Z-drug discontinuation | Primary/health/ social care<br>Secondary health/social care (not hospital)<br>Hospital (outpatient)<br>NR | Discontinuing treatment |

*Journal of Health Services Research & Policy*
**Optimising the prescribing of drugs that may cause dependency: an evidence and gap map of systematic reviews**
Shaw L, et al

| Focus/ Aim | Study (First author, date) | Medications of interest | Other medications included | Intervention name/s and aim | Outcomes measured | Setting/Context | Relevant part of care pathway |
|---|---|---|---|---|---|---|---|
| Evaluating intervention: deprescribing<br>Evaluating intervention: optimise prescribing | Furlan 2018 | Opiates | NA | Interventions to improve or ensure appropriate use of opioids: education, implementation of recommendations from clinical practice guidelines, using a tool to improve opioid prescribing, implementing urine drug tests, using disease management programs, reversal of overdose with naloxone, opioid substitution therapies, take-back program, prescription monitoring or review programs, regulations and policies, community or public health campaigns, collaborative strategies | Unintended consequences of implemented strategy, adverse consequences to participants or society, inappropriate use, misuse, abuse, addiction, overdose, deaths | Primary/health/ social care<br>Hospital (outpatient)<br>Patient home | Pre-treatment/ Initiation<br>Discontinuing treatment |

*Journal of Health Services Research & Policy*
**Optimising the prescribing of drugs that may cause dependency: an evidence and gap map of systematic reviews**
Shaw L, et al

| Focus/ Aim | Study (First author, date) | Medications of interest | Other medications included | Intervention name/s and aim | Outcomes measured | Setting/Context | Relevant part of care pathway |
|---|---|---|---|---|---|---|---|
| Evaluating intervention: deprescribing*. | Hart 2020 | Benzodiazepines<br>Opiates<br>Hypnotics/Z-drugs<br>Antidepressants | Antipsychotics, Antihistamines, Antivertigo, Anti-Parkinson, Urinary spasmolytics, Vasodilators, Digoxin, Diuretics, Beta Blockers, Hypoglycemics, Beta blocker eye drops, Alpha adrenergic antagonists, Alpha blockers, antihypertensives | Interventions to reduce falls or use of FRIDs: Face-to-face medication consultations with community pharmacy resident, fall-related assessment performed by research physician, geriatrician assessment of falls risk and systematic medication review, consideration of potential FRIDs for withdrawal+prescribing physician contact | Change in FRID use | Hospital (inpatient)<br>Hospital (outpatient) | Pre-treatment/ Initiation<br>Discontinuing treatment |
| Evaluating intervention: deprescribing<br>Evaluating intervention: optimise prescribing | Mauri 2020 | Opiates | NA | State opioid policies as a legislative or administrative action, such as a law or regulation, that directly targeted opioid misuse | Opioid prescribing and dispensing, patient behaviour, patient health | Primary/health/ social care<br>Hospital (inpatient)<br>Hospital (outpatient) | Pre-treatment/ Initiation<br>Maintaining treatment<br>Discontinuing treatment |

*Journal of Health Services Research & Policy*
**Optimising the prescribing of drugs that may cause dependency: an evidence and gap map of systematic reviews**
Shaw L, et al

| Focus/ Aim | Study (First author, date) | Medications of interest | Other medications included | Intervention name/s and aim | Outcomes measured | Setting/Context | Relevant part of care pathway |
|---|---|---|---|---|---|---|---|
| Evaluating intervention: enhance adherence | Chong 2011 | Antidepressants | NA | Interventions to improve antidepressant medication adherence: categories include: Educational focus, behavioural focus, Multifaceted interventions (more than a single component strategy (educational, behavioural or affective) targeting both the patient and the healthcare provider) | Depression, antidepressant medication adherence | Primary/health/social care<br>Hospital (outpatient) | Maintaining treatment |
| Evaluating intervention: enhance adherence | Hudson 2019 | Antidepressants | NA | Collaborative care including following components: Multidisciplinary approach, telephone delivered case management, structured evidence-based case management plan, scheduled and proactive patient follow-up, enhanced inter-professional communication/support. Aim: enhance medication adherence | Use of antidepressant medications, depressive symptoms | Primary/health/social care<br>Secondary health/social care (not hospital) | Maintaining treatment |

*Journal of Health Services Research & Policy*
**Optimising the prescribing of drugs that may cause dependency: an evidence and gap map of systematic reviews**
Shaw L, et al

| Focus/ Aim | Study (First author, date) | Medications of interest | Other medications included | Intervention name/s and aim | Outcomes measured | Setting/Context | Relevant part of care pathway |
|---|---|---|---|---|---|---|---|
| Evaluating intervention: enhance adherence | Polinski 2011 | Benzodiazepines<br>Antidepressants<br>Hypnotics/Z-drugs (sedatives) | HEDIS, high risk drugs, anti-convulsants, antihistamines, propoxyphene, digoxin, NSAIDs, doxazosin, muscle relaxants/anti-spasmodics, others, warfarin, oral anti-diabetics, antiretroviral medications, warfarin, clopidogrel, antipsychotics, proton pump inhibitors, antibiotics, statins, antihypertensives | Medicare Part D cost-sharing provisions and drug coverage rules. Aim: improve access to essential medications through reduced cost-sharing with focus on under and over-use of specific drugs and classes | Reduced out-of-pocket costs, health | Primary/health/social care<br>Hospital (outpatient) | Pre-treatment/ Initiation<br>Maintaining treatment<br>Discontinuing treatment |

*Journal of Health Services Research & Policy*
**Optimising the prescribing of drugs that may cause dependency: an evidence and gap map of systematic reviews**
Shaw L, et al

| Focus/ Aim | Study (First author, date) | Medications of interest | Other medications included | Intervention name/s and aim | Outcomes measured | Setting/Context | Relevant part of care pathway |
|---|---|---|---|---|---|---|---|
| Evaluating intervention: enhance adherence<br>Evaluating intervention: deprescribing | Dann-Reed 2020 | Benzodiazepines | Antipsychotics, anti-cholinergics, donepezil | Community pharmacy based intervention for people affected by dementia to optimise prescribing of medication (including medicine reconciliation, assessing for potentially inappropriate drugs). Interventions included: education, training Primary Care Navigators, pharmacist-led medication review, interdisciplinary educational programme, medication safety check, telephone call with patients and caregivers post discharge, inter-professional clinic, memory screening assessment, review of patients records, Donepezil outpatient consultation service | Willingness to pay, dementia severity, anticholinergic drug scale scores, number of falls/deaths, risk of falls, caregiver burden, patient satisfaction, number of prescribed medicine/patient, number of intervention, proportion discontinued psychotropics, proportion patients referred | Primary/health/social care<br>Secondary health/social care (not hospital)<br>Hospital (inpatient)<br>Hospital (outpatient)<br>Care home<br>Patient home | Pre-treatment/ Initiation<br>Maintaining treatment<br>Discontinuing treatment |

*Journal of Health Services Research & Policy*
**Optimising the prescribing of drugs that may cause dependency: an evidence and gap map of systematic reviews**
Shaw L, et al

| Focus/ Aim | Study (First author, date) | Medications of interest | Other medications included | Intervention name/s and aim | Outcomes measured | Setting/Context | Relevant part of care pathway |
|---|---|---|---|---|---|---|---|
| Evaluating intervention: optimise prescribing | Chhina 2013 | Benzodiazepines Antidepressants | Propoxyphene, cerebral & peripheral vasodilators, cephalexin, amoxycillin with or without clavulinic acid, cephalexin, doxycycline, erythromycin, penicillin, rrimethoprim, cefaclor, roxithromycin, NSAIDS, metronidazole, omeprazole, inhaled steroids, antipsychotic, antihypertensives | Academic Detailing to modify drug prescription behaviour of Family Physicians in primary care settings | Prescription rate | Primary/health/social care | Pre-treatment/ Initiation Maintaining treatment Discontinuing treatment |
| Evaluating intervention: optimise prescribing | Kunstler 2019 | Benzodiazepines Opiates Antidepressants | Antibiotics, COX-2, anti-inflammatories, beta-2-agonists, dietary supplements, antipsychotics | Interventions to change prescribing behaviour: educational outreach, academic detailing programs | Prescribing, quality assurance, safety | Primary/health/social care Secondary health/social care (not hospital) Patient home | Pre-treatment/ Initiation Discontinuing treatment |

*Journal of Health Services Research & Policy*
**Optimising the prescribing of drugs that may cause dependency: an evidence and gap map of systematic reviews**
Shaw L, et al

| Focus/ Aim | Study (First author, date) | Medications of interest | Other medications included | Intervention name/s and aim | Outcomes measured | Setting/Context | Relevant part of care pathway |
|---|---|---|---|---|---|---|---|
| Evaluating intervention: optimise prescribing | Loganathan 2011 | Benzodiazepines Hypnotics/Z-drugs | Neuroleptics, anti-psychotics, NSAIDs and paracetamol, psychotropics, warfarin, aspirin, antihypertensives | Interventions to optimise prescribing in care homes: staff education, multi-disciplinary team meetings, pharmacist medication reviews, computerised clinical decision support systems | Improvement in appropriate drug order | Care home | Pre-treatment/ Initiation Discontinuing treatment |
| Evaluating intervention: optimise prescribing | Weatherburn 2020 | Antidepressants | Coâproxamol, acetylcysteine, antidiabetics, antipsychotics, HRT, NSAIDs, combined oral contraceptive pill | Regulatory risk communication: ensure medication safety | Rate of prescribing. Secondary outcomes: rates of prescribing of substitute medicines, rates of prescribing of the target medicine in non-target population, change in intended/unintended health outcomes that were the focus of safety concern | NR | Pre-treatment/ Initiation Maintaining treatment |

Under Review

*Journal of Health Services Research & Policy*

**Optimising the prescribing of drugs that may cause dependency: an evidence and gap map of systematic reviews**

Shaw L, et al

| Focus/ Aim | Study (First author, date) | Medications of interest | Other medications included | Intervention name/s and aim | Outcomes measured | Setting/Context | Relevant part of care pathway |
|---|---|---|---|---|---|---|---|
| Evaluating intervention: optimise prescribing | Wetzel 2018 | Opiates | NA | Intervention associated with postsurgical prescribing: clinician mediated, patient mediated, organizational, behavioural | Discharge opioid prescriptions, quantity of opioids prescribed and used | Hospital (outpatient) | Pre-treatment/ Initiation |
| Evaluating intervention: optimise prescribing | Bourcier 2018 | Benzodiazepines<br>Antidepressants<br>Hypnotics/Z-drugs | First generation antihistamines | Regulatory or educational strategies to improve appropriate use of sedative-hypnotics in insomnia treatment | Changes in prescription and/or consumption of SH, switch to another non-recommended medication, changes in healthcare resource use, clinical adverse events | Primary/health/social care<br>Hospital (inpatient)<br>Hospital (outpatient)<br>Care home | Pre-treatment/ Initiation<br>Discontinuing treatment |
| Evaluating intervention: optimise prescribing | Nguyen 2020 | Benzodiazepines<br>Antidepressants | Antipsychotics, stimulants, teratogenic valproate, lithium monotherapy, methadone | Clinical Practice Guidelines on prescribing practice in mental health. Aim: change in mental health prescribing practice | Change in the direction of guideline recommendations | Primary/health/social care<br>Hospital (inpatient)<br>Hospital (outpatient) | Pre-treatment/ Initiation<br>Prescribing guidelines |

*Secondary aim or one of multiple aims; [a]Note that focus is on 'use' which includes initiating prescribing. Drugs of interest may be currently under- or over-used, [b]One aspect of broad aim, [c]One aspect of interventions. BP=Blood Pressure, CAD=Coronary Artery Disease, CPS=Community Pharmacist Services, CVD=Cardiovascular Disease, COX-2=Cyclooxygenase-2, DBP-Diastolic Blood Pressure, FDC=Fixed-Dose Combination, FRID=Fall Risk Increasing Drugs, HAART= Highly Active Antiretroviral Therapy, HBP=High Blood Pressure, HIV= Human Immunodeficiency Virus, HRT=Hormonal Replacement Therapy, HTS=Healthcare professional Targeted Services, INR=International Normalised Ratio, NA=Not Applicable, NR=Not Reported, NSAID=Non-Steroidal Anti-Inflammatories,PTS=Patient Targeted Services, SBP=Systolic Blood Pressure, SH=Sedative Hypnotics, SR=Sustained Release, QOL=Quality of Life,

*Journal of Health Services Research & Policy*
**Optimising the prescribing of drugs that may cause dependency: an evidence and gap map of systematic reviews**
Shaw L, et al

*Table 7: Systematic reviews synthesising studies conducting qualitative evidence synthesis*

| Study (First author, date) | Medications of interest | Other medications included | Perspectives obtained | Phenomenon of Interest | Setting/Context | Relevant part of care pathway |
|---|---|---|---|---|---|---|
| Anderson 2014 | Benzodiazepines<br>Opiates<br>Antidepressants<br>Hypnotics/Z-drugs | Psychotropics, proton pump inhibitors, miscellaneous PIMs | Practitioner/Prescriber | Prescribing<br>Experiences/views of an Intervention<br>(Deprescribing: *Perceptions related to stopping BZ in particular)* | Primary/health/social care<br>Secondary health/social care (not hospital)<br>Care home | Maintaining treatment<br>Discontinuing treatment |
| Kennedy 2019 | Opiates | NA | Practitioner/Prescriber | Prescribing | Primary/health/social care<br>Secondary health/social care (not hospital) | Pre-treatment/ Initiation<br>Maintaining treatment |

*Journal of Health Services Research & Policy*
**Optimising the prescribing of drugs that may cause dependency: an evidence and gap map of systematic reviews**
Shaw L, et al

| Study (First author, date) | Medications of interest | Other medications included | Perspectives obtained | Phenomenon of Interest | Setting/Context | Relevant part of care pathway |
|---|---|---|---|---|---|---|
| Toye 2017 | Opiates | NA | Practitioner/Prescriber | Prescribing | Primary/health/social care<br>Secondary health/social care (not hospital)<br>Hospital (outpatient) | Pre-treatment/ Initiation<br>Maintaining treatment<br>Discontinuing treatment |
| Cullinan 2014 | Benzodiazepines<br>Opiates<br>Antidepressants | Insulin, psychotropic medication | Practitioner/Prescriber | Prescribing | Primary/health/social care | Pre-treatment/ Initiation<br>Discontinuing treatment |

Under Review

*Journal of Health Services Research & Policy*

**Optimising the prescribing of drugs that may cause dependency: an evidence and gap map of systematic reviews**

Shaw L, et al

| Study (First author, date) | Medications of interest | Other medications included | Perspectives obtained | Phenomenon of Interest | Setting/Context | Relevant part of care pathway |
|---|---|---|---|---|---|---|
| Griffiths 2012 | Benzodiazepines<br>Hypnotics/Z-drugs<br>Antidepressants | Antipsychotics, lithium, atomoxetine, dexamphetamine, methylphenidat, stimulants for ADHD, lozapine | Practitioner/Prescriber<br>Patient | Prescribing | Prison<br>Hospital (inpatient)<br>*Forensic wards/hospitals* | Pre-treatment/ Initiation<br>Maintaining treatment<br>Discontinuing treatment |

*Journal of Health Services Research & Policy*

**Optimising the prescribing of drugs that may cause dependency: an evidence and gap map of systematic reviews**

Shaw L, et al

| Study (First author, date) | Medications of interest | Other medications included | Perspectives obtained | Phenomenon of Interest | Setting/Context | Relevant part of care pathway |
|---|---|---|---|---|---|---|
| Sirdifield 2017 | Benzodiazepines<br>Hypnotics/Z-drugs | NA | Patient | Reasons for adherence/non-adherence<br>Taking medication<br>Experiences/views of an intervention (deprescribing) | Primary/health/social care | Pre-treatment/ Initiation<br>Maintaining treatment<br>Discontinuing treatment |

*Journal of Health Services Research & Policy*
**Optimising the prescribing of drugs that may cause dependency: an evidence and gap map of systematic reviews**
Shaw L, et al

| Study (First author, date) | Medications of interest | Other medications included | Perspectives obtained | Phenomenon of Interest | Setting/Context | Relevant part of care pathway |
|---|---|---|---|---|---|---|
| Reeve 2013 | Benzodiazepines<br>Antidepressants | Any long-term medication, methadone (substitution program), antiepileptic's, HRT, PPIs, donepezil, antihypertensives | Patient<br>Carer | Experiences/views of an intervention (deprescribing) | NR | Discontinuing treatment |

*Journal of Health Services Research & Policy*
**Optimising the prescribing of drugs that may cause dependency: an evidence and gap map of systematic reviews**
Shaw L, et al

| Study (First author, date) | Medications of interest | Other medications included | Perspectives obtained | Phenomenon of Interest | Setting/Context | Relevant part of care pathway |
|---|---|---|---|---|---|---|
| Sirdifield 2013 | Benzodiazepines | NA | Practitioner/Prescriber | Prescribing<br>Experiences/views of an intervention (deprescribing) | Primary/health/social care | Pre-treatment/ Initiation<br>Discontinuing treatment |

*Journal of Health Services Research & Policy*
**Optimising the prescribing of drugs that may cause dependency: an evidence and gap map of systematic reviews**
Shaw L, et al

| Study (First author, date) | Medications of interest | Other medications included | Perspectives obtained | Phenomenon of Interest | Setting/Context | Relevant part of care pathway |
|---|---|---|---|---|---|---|
| Rashid 2018 | Antidepressants | Yes, relevant to the following conditions: asthma, renal transplant, proton pump inhibitors, schizophrenia, migraine, antiretroviral for HIV, osteoporosis, anxiety, malaria prophylaxis, bipolar, back pain, ADHD, dysphagia, IBD, RA, breast cancer, anticoagulants, borderline personality disorder,<br>Specific drugs mentioned: clozapine, sumatriptan, SSRI, HAART, malaria prophylaxis, antibiotics, phosphate binding, insulin, clopidogrel, PMTCT, chemotherapy, Aripiprazole, antihypertensives | Practitioner/Prescriber<br>Patient<br>Carer<br>Family member | Experiences/views of an intervention (adherence)<br>Taking a medication | Primary/health/social care<br>Secondary health/social care (not hospital)<br>Hospital (inpatient)<br>Hospital (outpatient) | Pre-treatment/ Initiation<br>Maintaining treatment |
| Ford 2017 | Antidepressants | NA | Practitioner/Prescriber | Prescribing<br>*(Secondary focus)* | Primary/health/social care | Pre-treatment/ Initiation |

der Review

*Journal of Health Services Research & Policy*
**Optimising the prescribing of drugs that may cause dependency: an evidence and gap map of systematic reviews**
Shaw L, et al

| Study (First author, date) | Medications of interest | Other medications included | Perspectives obtained | Phenomenon of Interest | Setting/Context | Relevant part of care pathway |
|---|---|---|---|---|---|---|
| Maund 2019a | Antidepressants | NA | Practitioner/Prescriber*<br>Patient | Prescribing*<br>Experiences/views of an intervention (deprescribing) | Primary/health/social care<br>Secondary health/social care (not hospital)<br>Hospital (inpatient)<br>Hospital (outpatient)<br>Care home | Discontinuing treatment |

* Data too thin for synthesis. Green highlighted text=High Overall Quality as appraised by AMSTAR-2, Turquoise highlighted text=Moderate Overall Quality as appraised by AMSTAR-2. ADHD=Attention-Deficit Hyperactivity Disorder, BZ=Benzodiazepine, HAART= Highly Active Antiretroviral Therapy, HIV= Human Immunodeficiency Virus, IBD=Irritable Bowel Disease, NA=Not Applicable, NR=Not Reported, PMTCT=Prevention of Mother-to-Child Transmission, RA=Rheumatoid Arthritis, SSRI=Selective Serotonin Reuptake Inhibitor

*Journal of Health Services Research & Policy*
**Optimising the prescribing of drugs that may cause dependency: an evidence and gap map of systematic reviews**
Shaw L, et al

*Table 8: Six systematic reviews which synthesised Clinical Practice Guidelines (CPGs)*

| Study (First author, date) | Medication of interest | Other medications included | Description | Setting |
|---|---|---|---|---|
| Dagenais 2010 | Opiates | NSAIDs, muscle relaxants, acetaminophen | Synthesis of recommendations for the assessment and management of low back pain from recent CPG | Primary/health/social care<br>Hospital (outpatient) |
| Ernstzen 2017 | Opiates | NA | Appraisal of available evidence-based CPGs for the management of chronic muscular skeletal pain | Primary/health/social care |
| Herzig 2018 | Opiates | NA | Synthesis of acute pain management guidelines for management of acute, non-cancer pain | Hospital (inpatient) |
| Mayer, 2010[125] | Opiates | Acetaminophen, NSAID, codeine, tramadol | Summarises recommendations from evidence-based CPGs for management of chronic lower back pain | Primary/health/social care<br>Secondary health/social care (not hospital) |
| Nuckols 2014 | Opiates | NA | Evaluation of quality and content of CPGs to: optimize patient care and use of opioids in the treatment of chronic pain:<br>dosing limits, medications and formulations, titration of dose, switching from one opioid to another, drug, drug interactions, drug-disease interactions, risk mitigation strategies (opioid risk assessment tools, written treatment agreements, and urine drug testing) | NR |
| Zhang, 2020a | Opiates | NA | Review recommendations on: prescription of opioids at discharge, appropriate disposal of opioids, prevention of chronic postsurgical opioid use after abdominopelvic surgery | Hospital (inpatient) |

Green highlighted text=High Overall Quality as appraised by the AMSTAR-2. CPG=Clinical Practice Guidelines, NA=Not Applicable

# References

1. Al Shemeili S. Exploring structures and processes of medicines management in elderly hospitalised patients in the United Arab Emirates [PhD]. OpenAIR@RGU: Robert Gordon University; 2015.
2. Al-Jumah KA and Qureshi NA. Impact of pharmacist interventions on patients' adherence to antidepressants and patient-reported outcomes: a systematic review. Patient Prefer Adherence 2012; 6: 87-100. DOI: https://doi.org/10.2147/PPA.S27436.
3. Anderson K, Stowasser D, Freeman C, et al. Prescriber barriers and enablers to minimising potentially inappropriate medications in adults: a systematic review and thematic synthesis. BMJ Open 2014; 4:e006544. DOI: https://doi.org/10.1136/bmjopen-2014-006544.
4. Awadalla R, Gnjidic D, Patanwala A, et al. The Effectiveness of Stewardship Interventions to Reduce the Prescribing of Extended-Release Opioids for Acute Pain: A Systematic Review. Pain Med 2020; 21: 2401-11. DOI: https://doi.org/10.1093/pm/pnaa139.
5. Baandrup L, Ebdrup BH, Rasmussen JO, et al. Pharmacological interventions for benzodiazepine discontinuation in chronic benzodiazepine users. Cochrane Database Syst Rev 2018; 3: CD011481. DOI: https://doi.org/10.1002/14651858.CD011481.pub2.
6. Beaudoin FL, Banerjee GN and Mello MJ. State-level and system-level opioid prescribing policies: The impact on provider practices and overdose deaths, a systematic review. J Opioid Manag 2016; 12: 109-18. https://doi.org/10.5055/jom.2016.0322.
7. Becker SJ and Midoun MM. Effects of Direct-To-Consumer Advertising on Patient Prescription Requests and Physician Prescribing: A Systematic Review of Psychiatry-Relevant Studies. J Clin Psychiatry 2016; 77: e1293-e300. DOI: https://doi.org/10.4088/JCP.15r10325.
8. Bourcier E, Korb-Savoldelli V, Hejblum G, et al. A systematic review of regulatory and educational interventions to reduce the burden associated with the prescriptions of sedative-hypnotics in adults treated for sleep disorders. PLoS One 2018; 13:e0191211. DOI: https://doi.org/10.1371/journal.pone.0191211.
9. Brown JVE, Walton N, Meader N, Todd A, Webster LA, Steele R, et al. Pharmacy-based management for depression in adults. Cochrane Database Syst Rev 2019;12:CD013299. DOI: https://doi.org/10.1002/14651858.CD013299.pub2
10. Chhina HK, Bhole VM, Goldsmith C, et al. Effectiveness of academic detailing to optimize medication prescribing behaviour of family physicians. J Pharm Pharm Sci 2013; 16: 511-29. DOI: https://doi.org/10.18433/j3kk6c.
11. Chong WW, Aslani P and Chen TF. Effectiveness of interventions to improve antidepressant medication adherence: a systematic review. Int J Clin Pract 2011; 65: 954-75. DOI: https://doi.org/10.1111/j.1742-1241.2011.02746.x
12. Conn VS, Ruppar TM, Enriquez M, et al. Medication adherence interventions that target subjects with adherence problems: Systematic review and meta-analysis. Res Social Adm Pharm 2016; 12: 218-46. DOI: https://doi.org/10.1016/j.sapharm.2015.06.001.
13. Coronado-Vázquez V, Canet-Fajas C, Delgado-Marroquín MT, Magallón-Botaya R, Romero-Martín M, Gómez-Salgado J. Interventions to facilitate shared decision-making using decision aids with patients in Primary Health Care: A systematic review. Medicine (Baltimore). 2020 Aug 7;99(32):e21389. DOI: 10.1097/MD.0000000000021389.

Under Review

14. Cullinan S, O'Mahony D, Fleming A, et al. A meta-synthesis of potentially inappropriate prescribing in older patients. Drugs Aging 2014: 31: 631-8. DOI: https://doi.org/10.1007/s40266-014-0190-4.
15. Dagenais S, Tricco AC and Haldeman S. Synthesis of recommendations for the assessment and management of low back pain from recent clinical practice guidelines. Spine J 2010; 10: 514-29. DOI: https://doi.org/10.1016/j.spinee.2010.03.032.
16. Dann-Reed E, Poland F and Wright D. Systematic review to inform the development of a community pharmacy-based intervention for people affected by dementia. Int J Pharm Pract 2020; 28: 233-45. DOI: https://doi.org/10.1111/ijpp.12586.
17. Darker CD, Sweeney BP, Barry JM, et al. Psychosocial interventions for benzodiazepine harmful use, abuse or dependence. Cochrane Database Syst Rev 2015; DOI: https://doi.org/10.1002/14651858.CD009652.pub2.
18. Dills H, Shah K, Messinger-Rapport B, et al. Deprescribing Medications for Chronic Diseases Management in Primary Care Settings: A Systematic Review of Randomized Controlled Trials. J Am Med Dir Assoc 2018; 19: 923-35 e2. DOI: https://doi.org/10.1016/j.jamda.2018.06.021.
19. Eccleston C, Fisher E, Thomas KH, et al. Interventions for the reduction of prescribed opioid use in chronic non-cancer pain. Cochrane Database Syst Rev 2017; 11: CD010323. DOI: https://doi.org/10.1002/14651858.CD010323.pub3.
20. Ernstzen DV, Louw QA and Hillier SL. Clinical practice guidelines for the management of chronic musculoskeletal pain in primary healthcare: a systematic review. Implement Sci 2017; 12: 1. DOI: https://doi.org/10.1186/s13012-016-0533-0.
21. Ford E, Lee S, Shakespeare J and Ayers S. Diagnosis and management of perinatal depression and anxiety in general practice: a meta-synthesis of qualitative studies. Br J Gen Pract 2017; 67: e538-e46. DOI: https://doi.org/10.3399/bjgp17X691889.
22. Frank JW, Lovejoy TI, Becker WC, et al. Patient Outcomes in Dose Reduction or Discontinuation of Long-Term Opioid Therapy: A Systematic Review. Ann Intern Med 2017; 167: 181-91. DOI: https://doi.org/10.7326/M17-0598.
23. Furlan AD, Carnide N, Irvin E, et al. A systematic review of strategies to improve appropriate use of opioids and to reduce opioid use disorder and deaths from prescription opioids. Canadian Journal of Pain 2018; 2: 218-35. DOI: https://doi.org/10.1080/24740527.2018.1479842.
24. Garcia-Perez L and Serrano-Aguilar P. Cost-effectiveness of interventions to enhance medication adherence in psychiatric patients: a systematic review. Curr Clin Pharmacol 2011; 6: 115-24. DOI: https://doi.org/10.2174/157488411796151147.
25. Garcia-Perez L, Linertova R, Serrano-Perez P, et al. Interventions to improve medication adherence in mental health: the update of a systematic review of cost-effectiveness. Int J Psychiatry Clin Pract 2020; 24: 416-27. DOI: https://doi.org/10.1080/13651501.2020.1782434.
26. Gillaizeau F, Chan E, Trinquart L, et al. Computerized advice on drug dosage to improve prescribing practice. Cochrane Database Syst Rev 2013; DOI: 10.1002/14651858.CD002894.pub3:CD002894.
27. Gould RL, Coulson MC, Patel N, et al Interventions for reducing benzodiazepine use in older people: meta-analysis of randomised controlled trials. Br J Psychiatry 2014; 204: 98-107. DOI: https://doi.org/10.1192/bjp.bp.113.126003.


Under Review

28. Griffiths EV, Willis J and Spark MJ. A systematic review of psychotropic drug prescribing for prisoners. Aust N Z J Psychiatry 2012; 46: 407-21. DOI: https://doi.org/10.1177/0004867411433893.
29. Hart LA, Phelan EA, Yi JY, et al. Use of Fall Risk-Increasing Drugs Around a Fall-Related Injury in Older Adults: A Systematic Review. J Am Geriatr Soc 2020; 68: 1334-43. DOI: https://doi.org/10.1111/jgs.16369.
30. Herzig SJ, Calcaterra SL, Mosher HJ, et al. Safe Opioid Prescribing for Acute Noncancer Pain in Hospitalized Adults: A Systematic Review of Existing Guidelines. J Hosp Med 2018; 13: 256-62. DOI: https://doi.org/10.12788/jhm.2979.
31. Hopkins RE, Bui T, Magliano D, et al. Prescriber Education Interventions to Optimize Opioid Prescribing in Acute Care: A Systematic Review. Pain Physician 2019; 22: E551-E62.
32. Hoyle DJ, Bindoff IK, Clinnick L et al. Clinical and Economic Outcomes of Interventions to Reduce Antipsychotic and Benzodiazepine Use Within Nursing Homes: A Systematic Review. Drugs Aging 2018; 35: 123-34. DOI: https://doi.org/10.1007/s40266-018-0518-6.
33. Huang Y, Wei X, Wu T, et al.. Collaborative care for patients with depression and diabetes mellitus: a systematic review and meta-analysis. BMC Psychiatry 2013; 13: 260. DOI: https://doi.org/10.1186/1471-244X-13-260.
34. Hudson JL, Bower P, Kontopantelis E, et al. Impact of telephone delivered case-management on the effectiveness of collaborative care for depression and anti-depressant use: A systematic review and meta-regression. PLoS One 2019; 14:e0217948. DOI: https://doi.org/10.1371/journal.pone.0217948.
35. Hukins D, Macleod U and Boland JW. Identifying potentially inappropriate prescribing in older people with dementia: a systematic review. Eur J Clin Pharmacol 2019; 75: 467-81. DOI: https://doi.org/10.1007/s00228-018-02612-x.
36. Kennedy MC, Pallotti P, Dickinson R, et al. 'If you can't see a dilemma in this situation you should probably regard it as a warning': a metasynthesis and theoretical modelling of general practitioners' opioid prescription experiences in primary care. Br J Pain 2019; 13: 159-76.DOI: https://doi.org/10.1177/2049463718804572.
37. Kimmel HJ, Brice YN, Trikalinos TA, et al. Real-Time Emergency Department Electronic Notifications Regarding High-Risk Patients: A Systematic Review. Telemed J E Health 2019; 25: 604-18. DOI: https://doi.org/10.1089/tmj.2018.0117.
38. King S, Miani C, Exley J, et al. Impact of issuing longer- versus shorter-duration prescriptions: a systematic review. Br J Gen Pract 2018; 68: e286-e92. DOI: https://doi.org/10.3399/bjgp18X695501.
39. Kunstler BE, Lennox A and Bragge P. Changing prescribing behaviours with educational outreach: an overview of evidence and practice. BMC Med Educ 2019; 19: 311. DOI: https://doi.org/10.1186/s12909-019-1735-3.
40. Loganathan M, Singh S, Franklin BD, et al. Interventions to optimise prescribing in care homes: systematic review. Age Ageing 2011; 40: 150-62. https://doi.org/10.1093/ageing/afq161.
41. Low LF, Fletcher J, Goodenough B, et al. A Systematic Review of Interventions to Change Staff Care Practices in Order to Improve Resident Outcomes in Nursing Homes. PLoS One 2015; 10:e0140711. DOI: https://doi.org/10.1371/journal.pone.0140711.


Under Review

*Journal of Health Services Research & Policy*
**Optimising the prescribing of drugs that may cause dependency: an evidence and gap map of systematic reviews**
Shaw L, et al


42. Lynch T, Ryan C, Hughes CM, et al. Brief interventions targeting long-term benzodiazepine and Z-drug use in primary care: a systematic review and meta-analysis. Addiction 2020; 115: 1618-39. DOI: https://doi.org/10.1111/add.14981.
43. Mathieson S, Maher CG, Ferreira GE, et al. Deprescribing Opioids in Chronic Non-cancer Pain: Systematic Review of Randomised Trials. Drugs 2020; 80: 1563-76. DOI: https://doi.org/10.1007/s40265-020-01368-y.
44. Maund E, Dewar-Haggart R, Williams S, et al. Barriers and facilitators to discontinuing antidepressant use: A systematic review and thematic synthesis. J Affect Disord 2019a; 245: 38-62. DOI: https://doi.org/10.1016/j.jad.2018.10.107.
45. Maund E, Stuart B, Moore M, et al. Managing Antidepressant Discontinuation: A Systematic Review. Ann Fam Med 2019b; 17: 52-60. DOI: https://doi.org/10.1370/afm.2336.
46. Mauri AI, Townsend TN and Haffajee RL. The Association of State Opioid Misuse Prevention Policies With Patient- and Provider-Related Outcomes: A Scoping Review. Milbank Q 2020; 98: 57-105. https://doi.org/10.1111/1468-0009.12436.
47. Mayer JM, Haldeman S, Tricco AC, et al. Management of chronic low back pain in active individuals. Curr Sports Med Rep 2010; 9: 60-6. DOI: https://doi.org/10.1249/JSR.0b013e3181caa9b6.
48. Milosavljevic A, Aspden T and Harrison J. Community pharmacist-led interventions and their impact on patients' medication adherence and other health outcomes: a systematic review. Int J Pharm Pract 2018; 26: 387-97. DOI: https://doi.org/10.1111/ijpp.12462.
49. Moise N, Falzon L, Obi M, et al. Interventions to Increase Depression Treatment Initiation in Primary Care Patients: a Systematic Review. J Gen Intern Med 2018; 33: 1978-89. DOI: https://doi.org/10.1007/s11606-018-4554-z.
50. Mokhar A, Topp J, Harter M, et al. Patient-centered care interventions to reduce the inappropriate prescription and use of benzodiazepines and z-drugs: a systematic review. PeerJ 2018; 6: e5535. DOI: https://doi.org/10.7717/peerj.5535.
51. Morera-Fumero AL, Fernandez-Lopez L and Abreu-Gonzalez P. Melatonin and melatonin agonists as treatments for benzodiazepines and hypnotics withdrawal in patients with primary insomnia. A systematic review. Drug Alcohol Depend 2020; 212: 107994. DOI: https://doi.org/10.1016/j.drugalcdep.2020.107994.
52. Mugunthan K, McGuire T and Glasziou P. Minimal interventions to decrease long-term use of benzodiazepines in primary care: a systematic review and meta-analysis. Br J Gen Pract 2011; 61: e573-8. DOI: https://doi.org/10.3399/bjgp11X593857.
53. Nguyen T, Seiler N, Brown E, et al. The effect of Clinical Practice Guidelines on prescribing practice in mental health: A systematic review. Psychiatry Res 2020; 284:112671. DOI: https://doi.org/10.1016/j.psychres.2019.112671.
54. Nieuwlaat R, Wilczynski N, Navarro T, et al. Interventions for enhancing medication adherence. Cochrane Database Syst Rev 2014; DOI https://doi.org/10.1002/14651858.CD000011.pub4.
55. Nuckols TK, Anderson L, Popescu I, et al. Opioid prescribing: a systematic review and critical appraisal of guidelines for chronic pain. Ann Intern Med 2014; 160: 38-47. DOI: https://doi.org/10.7326/0003-4819-160-1-201401070-00732.
56. Nussbaumer B, Morgan LC, Reichenpfader U, et al. Comparative efficacy and risk of harms of immediate- versus extended-release second-generation antidepressants: a systematic

*Journal of Health Services Research & Policy*
**Optimising the prescribing of drugs that may cause dependency: an evidence and gap map of systematic reviews**
Shaw L, et al

review with network meta-analysis. CNS Drugs 2014; 28: 699-712. DOI: https://doi.org/10.1007/s40263-014-0169-z.

57. Ostini R, Jackson C, Hegney D, et al. How is medication prescribing ceased? A systematic review. Med Care 2011; 49: 24-36. DOI: https://doi.org/10.1097/MLR.0b013e3181ef9a7e.
58. Page AT, Clifford RM, Potter K, et al. The feasibility and effect of deprescribing in older adults on mortality and health: a systematic review and meta-analysis. Br J Clin Pharmacol 2016; 82: 583-623. DOI: https://doi.org/10.1111/bcp.12975.
59. Pantoja T, Grimshaw JM, Colomer N, et al. Manually-generated reminders delivered on paper: effects on professional practice and patient outcomes. Cochrane Database Syst Rev 2019; 12: CD001174. DOI: https://doi.org/10.1002/14651858.CD001174.pub4.
60. Park YJ and Martin EG. Medicare Part D's Effects on Drug Utilization and Out-of-Pocket Costs: A Systematic Review. Health Serv Res 2017; 52: 1685-728. DOI: https://doi.org/10.1111/1475-6773.12534.
61. Polinski JM, Donohue JM, Kilabuk E, et al. Medicare Part D's effect on the under- and overuse of medications: a systematic review. J Am Geriatr Soc 2011; 59: 1922-33. DOI: https://doi.org/10.1111/j.1532-5415.2011.03537.x.
62. Pollmann AS, Murphy AL, Bergman JC, et al. Deprescribing benzodiazepines and Z-drugs in community-dwelling adults: a scoping review. BMC Pharmacol Toxicol 2015; 16: 19. DOI: https://doi.org/10.1186/s40360-015-0019-8.
63. Posadzki P, Mastellos N, Ryan R, et al. Automated telephone communication systems for preventive healthcare and management of long-term conditions. Cochrane Database Syst Rev 2016; 12: CD009921. DOI: https://doi.org/10.1002/14651858.CD009921.pub2.
64. Rashid MA, Llanwarne N, Heyns N, et al. What are the implications for practice that arise from studies of medication taking? A systematic review of qualitative research. PLoS One 2018; 13: e0195076. DOI: https://doi.org/10.1371/journal.pone.0195076.
65. Readdean KC, Heuer AJ and Scott Parrott J. Effect of pharmacist intervention on improving antidepressant medication adherence and depression symptomology: A systematic review and meta-analysis. Res Social Adm Pharm 2018; 14: 321-31. DOI: https://doi.org/10.1016/j.sapharm.2017.05.008.
66. Reeve E, Ong M, Wu A, et al. A systematic review of interventions to deprescribe benzodiazepines and other hypnotics among older people. Eur J Clin Pharmacol 2017; 73: 927-35. DOI: https://doi.org/10.1007/s00228-017-2257-8.
67. Reeve E, To J, Hendrix I, et al. Patient barriers to and enablers of deprescribing: a systematic review. Drugs Aging 2013; 30: 793-807. DOI: https://doi.org/10.1007/s40266-013-0106-8.
68. Rubio-Valera M, Serrano-Blanco A, Magdalena-Belio J, et al. Effectiveness of pharmacist care in the improvement of adherence to antidepressants: a systematic review and meta-analysis. Ann Pharmacother 2011; 45: 39-48. DOI: https://doi.org/10.1345/aph.1P429.
69. Sirdifield C, Anthierens S, Creupelandt H, et al. General practitioners' experiences and perceptions of benzodiazepine prescribing: systematic review and meta-synthesis. BMC Fam Pract 2013; 14: 191. DOI: https://doi.org/10.1186/1471-2296-14-191.
70. Sirdifield C, Chipchase SY, Owen S, et al. A Systematic Review and Meta-Synthesis of Patients' Experiences and Perceptions of Seeking and Using Benzodiazepines and Z-Drugs: Towards Safer Prescribing. Patient 2017; 10: 1-15. DOI: https://doi.org/10.1007/s40271-016-0182-z.

Under Review

*Journal of Health Services Research & Policy*
**Optimising the prescribing of drugs that may cause dependency: an evidence and gap map of systematic reviews**
Shaw L, et al


71. Tedesco D, Gori D, Desai KR, et al. Drug-Free Interventions to Reduce Pain or Opioid Consumption After Total Knee Arthroplasty: A Systematic Review and Meta-analysis. JAMA Surg 2017; 152: e172872. DOI: https://doi.org/10.1001/jamasurg.2017.2872.
72. Toye F, Seers K, Tierney S, et al. A qualitative evidence synthesis to explore healthcare professionals' experience of prescribing opioids to adults with chronic non-malignant pain. BMC Fam Pract 2017; 18: 94. DOI: https://doi.org/10.1186/s12875-017-0663-8.
73. Weatherburn CJ, Guthrie B, Dreischulte T, et al. Impact of medicines regulatory risk communications in the UK on prescribing and clinical outcomes: Systematic review, time series analysis and meta-analysis. Br J Clin Pharmacol 2020; 86: 698-710. DOI: https://doi.org/10.1111/bcp.14104.
74. Wetzel M, Hockenberry J and Raval MV. Interventions for Postsurgical Opioid Prescribing: A Systematic Review. JAMA Surg 2018; 153: 948-54. DOI: https://doi.org/10.1001/jamasurg.2018.2730.
75. Wilbur K, Elmubark A and Shabana S. Systematic Review of Standardized Patient Use in Continuing Medical Education. J Contin Educ Health Prof 2018; 38: 3-10. DOI: https://doi.org/10.1097/CEH.0000000000000190.
76. Wilson MN, Hayden JA, Rhodes E, et al. Effectiveness of Prescription Monitoring Programs in Reducing Opioid Prescribing, Dispensing, and Use Outcomes: A Systematic Review. Journal of Pain 2019; 20: 1383-93. DOI: https://doi.org/10.1016/j.jpain.2019.04.007.
77. Wong CK, O'Rielly CM, Teitge BD, et al. The Characteristics and Effectiveness of Interventions for Frequent Emergency Department Utilizing Patients With Chronic Noncancer Pain: A Systematic Review. Acad Emerg Med 2020; 27: 742-52. DOI: https://doi.org/10.1111/acem.13934.
78. Xie CX, Chen Q, Hincapié CA, Hofstetter L, Maher CG, Machado GC. Effectiveness of clinical dashboards as audit and feedback or clinical decision support tools on medication use and test ordering: a systematic review of randomized controlled trials. J Am Med Inform Assoc. 2022 Sep 12;29(10):1773-1785. DOI: 10.1093/jamia/ocac094.
79. Zhang DDQ, Dossa F, Arora A, et al. Recommendations for the Prescription of Opioids at Discharge After Abdominopelvic Surgery: A Systematic Review. JAMA Surg 2020a; 155:420-9. DOI: https://doi.org/10.1001/jamasurg.2019.5875.
80. Zhang DDQ, Sussman J, Dossa F, et al. A Systematic Review of Behavioral Interventions to Decrease Opioid Prescribing After Surgery. Ann Surg 2020b; 271:266-78. DOI: https://doi.org/10.1097/SLA.0000000000003483.


Under Review
